# Bridging Plastic Recycling and Organic Catalysis: Photocatalytic Deconstruction of Polystyrene via a C–H Oxidation Pathway

**DOI:** 10.1021/acscatal.2c02292

**Published:** 2022-06-23

**Authors:** Tengfei Li, Arjun Vijeta, Carla Casadevall, Alexander S. Gentleman, Tijmen Euser, Erwin Reisner

**Affiliations:** † Yusuf Hamied Department of Chemistry, University of Cambridge, Cambridge CB2 1EW, U.K.; ‡ Department of Natural Sciences, Manchester Metropolitan University, Manchester M1 5GD, U.K.; § Cavendish Laboratory, University of Cambridge, Cambridge CB3 0HE, U.K.

**Keywords:** plastic deconstruction, polystyrene, benzoic acid, photocatalysis, C−H oxidation

## Abstract

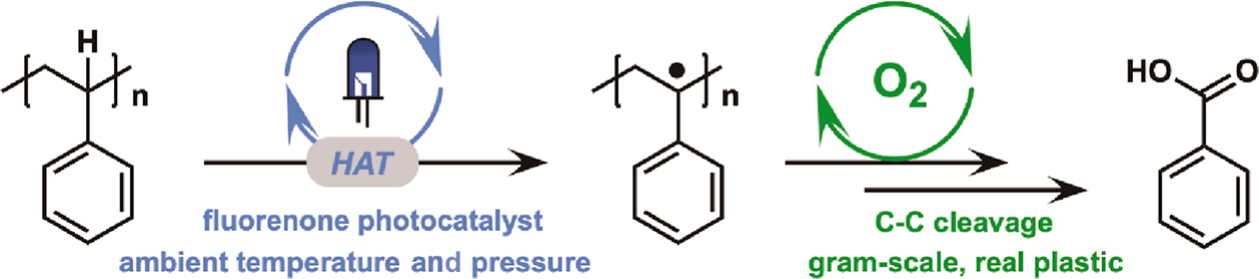

Chemical recycling of synthetic polymers represents a promising strategy to deconstruct plastic waste and make valuable products. Inspired by small-molecule C–H bond activation, a visible-light-driven reaction is developed to deconstruct polystyrene (PS) into ∼40% benzoic acid as well as ∼20% other monomeric aromatic products at 50 °C and ambient pressure. The practicality of this strategy is demonstrated by deconstruction of real-world PS foam on a gram scale. The reaction is proposed to proceed via a C–H bond oxidation pathway, which is supported by theoretical calculations and experimental results. Fluorescence quenching experiments also support efficient electron transfer between the photocatalyst and the polymer substrate, providing further evidence for the proposed mechanism. This study introduces concepts from small-molecule catalysis to polymer deconstruction and provides a promising method to tackle the global crisis of plastic pollution.

## Introduction

**Figure 1:**
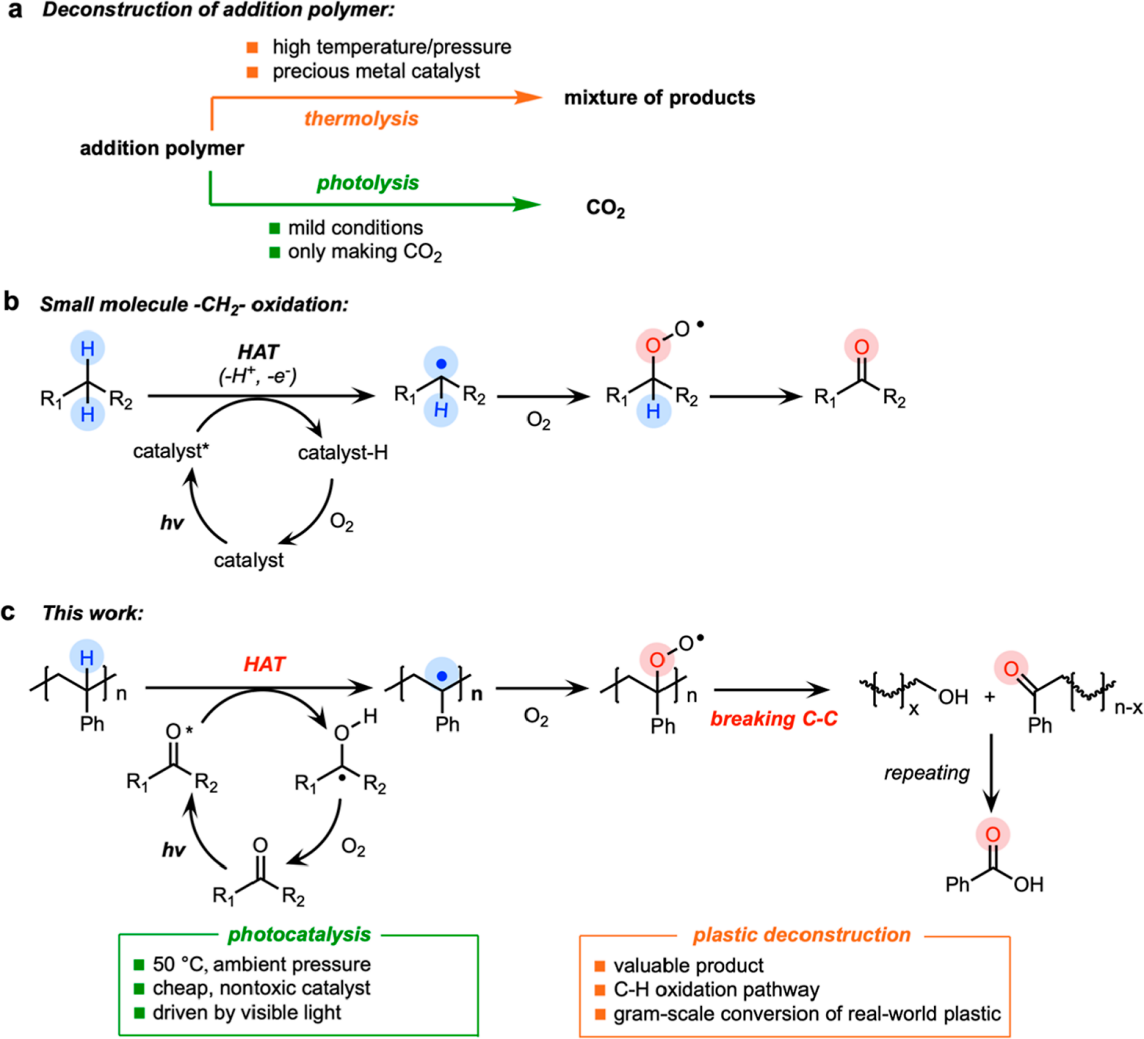
Deconstruction of addition polymers by C–H oxidation. (a) Summary of previous reports and (b) prior art on small-molecule −CH_2_– oxidation mediated by a HAT photocatalyst. (c) Schematic illustration of the photocatalytic deconstruction of an addition polymer through C–H oxidation on a tertiary sp^3^ carbon in this work. The deconstruction of PS into benzoic acid is demonstrated with an aromatic ketone as the HAT photocatalyst.

More than 8 billion tons of plastic have been produced since 1950, over 80% of which has become waste in landfills or escaped into the environment.^1,2^ Plastic pollution is not only a serious health threat to animals and humans^3^ but also a loss of valuable chemical resources, thus providing the impetus to develop sustainable strategies to deconstruct plastic waste into useful chemical feedstocks. Addition polymers, such as polyethylene (PE), polypropylene (PP), and polystyrene (PS), account for >60% of plastic waste,^1^ and their controlled deconstruction is particularly challenging due to their inertness and the stability of the non-polar C–C bonds in the polymer backbone.^4−6^ Existing methods for the deconstruction of addition polymers are typically based on thermal processes, which require high temperatures, high pressures, and/or precious metal catalysts (Figure 1a).^7−11^ For example, Partenheimer showed that PE, PP, and PS can be converted to organic acids under 200 °C and 70 bar with metal/bromide catalysts.^12^ Edwards et al. demonstrated that strong microwave irradiation on an FeAlO_*x*_ catalyst can create a high local temperature and drive the deconstruction of PE, PP, and PS into H_2_ and carbon nanotubes within minutes.^13^ Perras and co-workers developed a hydrogenolysis system to transform PE into alkanes using a Pt–SiO_2_ catalyst under 300 °C and 1.4 MPa H_2_.^14^ Scott’s group coupled hydrogenolysis with aromatization to convert PE into a mixture of aromatic products with a Pt–Al_2_O_3_ catalyst at 280 °C.^15^ There are also recent reports on mechanical milling depolymerization of PS into styrene.^16^

The energized charge carriers created by photosensitizers offer a sustainable method to utilize light to drive addition polymer deconstruction under mild conditions (Figure 1a). In 1981, Kawai and Sakata reported the first photoconversion of PE, polyvinyl chloride (PVC), and polyvinyl alcohol into CO_2_ and H_2_ with a Pt–TiO_2_ catalyst under ultraviolet (UV) light.^17^ Soo’s group developed a vanadium complex photocatalyst that can decompose polyethylene-*block*-polyethylene glycol and hydroxyl-terminated PE into CO_2_ [small amounts of formic acid (∼5%) were also formed after a 1 week reaction], where the terminal hydroxyl group is believed to induce the depolymerization.^18^ Recently, Xie’s group demonstrated that sunlight and a Nb_2_O_5_ photocatalyst can drive the oxidative degradation of PE, PP, and PVC into CO_2_, which was further converted to acetic acid by photoreduction on the same catalyst.^19^ The reported photocatalysts are capable of driving the deconstruction of addition polymers under mild conditions. However, the only carbon product that can be generated in these photoconversion processes from oxidation is CO_2_, which is a greenhouse gas and has limited economic value. Photocatalytic deconstruction processes that can produce valuable organics (instead of CO_2_) from addition polymers are therefore desirable.

The bond cleavage chemistry of small hydrocarbon molecules can provide inspiration for addition polymer deconstruction. The activation of stable C–H bonds and achieving selective chemistry are the major challenge in C–H bond oxidation,^20−23^ which can be addressed by employing a hydrogen atom transfer (HAT) catalyst.^24−27^ The HAT process generates an organic radical when mediating between the excited state of the photocatalyst and the hydrocarbon substrate (Figure 1b). This radical reacts with O_2_ to give an organic peroxyl radical intermediate, and the second hydrogen is then abstracted from the organic intermediate to form an oxygenated product. Overall, this process can break two C–H bonds of a secondary sp^3^ carbon (−CH_2_−) and form a carbonyl (C=O) group. Reports on thermal C–H oxidation of a tertiary sp^3^ carbon (only one hydrogen on the carbon) that can break the adjacent C–C bond (instead of a C–H bond) are available,^28^ but the process is not catalytic and requires high temperatures (150–200 °C). Recent reports also show that polyolefins (e.g., PE and PP) can be modified by C–H functionalization to form functionalized polymers containing oxygenated groups,^29−31^ indicating that polymer reactions can be performed and studied in a way similar to that of small molecules.

Inspired by the above reports, we introduce herein a plastic deconstruction method through photocatalytic C–H oxidation of the tertiary carbons in the polymer backbone (Figure 1c). Recent reports have demonstrated the feasibility of driving photocatalytic deconstruction of PS with FeCl_3_ or acid.^32−34^ In our report, the photocatalysis is driven by a blue LED under mild temperature and pressure (50 °C, 1 bar, aerobic O_2_) with an aromatic ketone as the HAT photocatalyst. The HAT catalytic cycle and the formation of the peroxyl radical intermediate are comparable to small-molecule −CH_2_– oxidation, but the absence of the second hydrogen induces the cleavage of the C–C bonds in the polymer backbone to give the degradation products. PS, a widely used polymer that accounts for ∼10% of global plastic waste generation,^1^ is deconstructed to form benzoic acid (a useful chemical that serves as precursor to phenols, plasticizers, and food preservatives) with ∼40% yield as well as other identified monomeric aromatic products with ∼20% yield. We also demonstrate gram-scale deconstruction of PS and real-world PS foam. A reaction pathway involving C–H oxidation is proposed, which is supported by experimental and theoretical results. The electron transfer between the excited state of the photocatalyst and the polymer substrate is confirmed by Stern–Volmer experiments, providing further evidence for the proposed mechanism. This work does not only connect small-molecule catalysis with polymer deconstruction but also provides opportunities for plastic recycling industries to produce useful chemical feedstocks from plastic waste under ambient temperature and pressure.

## Results and Discussion

### Photocatalytic Deconstruction of PS

The PS starting material has a weight-average molecular weight (*M*_w_) of around 260 000 and a polydispersity index (PDI) of around 2.5. The homogeneous PS solution was first prepared by dissolving 20.8 mg of PS (0.2 mmol, the amount of polymer is considered as the mole amount of the styrene monomer, see eq S1) in 2 mL of ethyl acetate (EtOAc) solvent, giving a homogeneous solution containing 0.1 M PS. Fluorenone (a HAT C–H oxidation photocatalyst)^24,35^ and H_2_SO_4_ were added to the polymer solution. Photocatalysis was performed under blue LED irradiation (λ_max_ ∼ 450 nm, emission spectrum shown in Figure S1) and oxygen was provided by an O_2_ balloon (photoreactor shown in Figure S2). The blue LED (14.4 W) created a local temperature of around 50 ± 3 °C for the reaction. The product yield was determined using a quantitative high-performance liquid chromatograph (HPLC, equipped with UV−vis and mass spectrometry detectors) and a proton nuclear magnetic resonance (^1^H NMR) spectroscope. The *M*_w_ of the reaction mixture was analyzed by gel permeation chromatography (GPC). More details of the experimental setup can be found in the Supporting Information.

**Figure 2:**
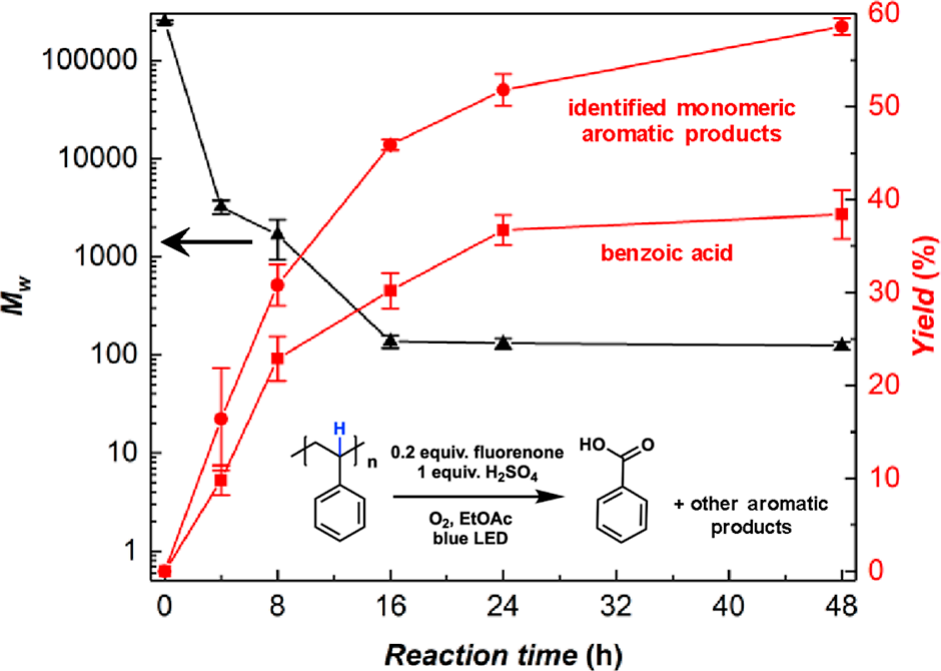
Photocatalytic deconstruction of PS. Changes of the weight-averaged molecular weight (*M*_w_, black; measured by GPC) of PS and the yields (red; measured by ^1^H NMR and HPLC) of all identified monomeric aromatic products and benzoic acid over the reaction time. Reaction conditions: 0.1 M PS, 0.2 equiv of fluorenone, 1 equiv of H_2_SO_4_, 2 mL of EtOAc, O_2_ balloon, blue LED, 50 ± 3 °C (due to the heating from the LED). The product yield is calculated by the product concentration divided by the initial concentration of the substrate (0.1 M). Error bars correspond to the standard deviation of triplicate experiments. The remaining ∼40% yield of aromatic products at 48 h are aromatic oligomers.

As shown in Figure 2, PS was deconstructed to small-molecule aromatic products. The major product was benzoic acid, with yields of 30 ± 2% at 16 h and 38 ± 3% at 48 h, respectively [^1^H NMR spectra and HPLC traces shown in Figures S3–S5]. The M_w_ of PS decreased from 260,000 to 3,200 (4 h) and 1,700 (8 h). No GPC peak for PS was observed after 16 h when the *M*_w_ stabilized between 100 and 200 (GPC traces and *M*_w_ results are shown in Figure S6). Small amounts of other monomeric aromatic products, such as ethyl benzoate (∼10%), acetophenone (∼6%), and benzaldehyde (∼1%), were also detected by ^1^H NMR spectroscopy and the mass spectrometry detector of HPLC. Gas chromatography-mass spectroscopy (GC–MS) also confirms the formation of ethyl benzoate as well as phenylglyoxylic acid, another aromatic byproduct (Figure S7). The overall yield of all identified monomeric aromatic products (benzoic acid, ethyl benzoate, acetophenone, benzaldehyde, and phenylglyoxylic acid) at 48 h was ∼60%. A mass balance of 100% was confirmed by the integration of the aromatic region in the NMR spectrum (Figure S8). The remaining ∼40% yield of aromatic products are aromatic functionalized oligomers with a molecular weight between 200 and 800, as shown in the MS results in Figure S9. The formation of stable functionalized oligomers is also consistent with previous reports.^33^ Gaseous CO (21% yield) and CO_2_ (7.5% yield) were detected in the headspace of the reactor after 16 h by gas chromatography (Figures S10 and S11). These gases arose from decarboxylation originating from the methylene groups in PS. The majority (∼95%) of the fluorenone photocatalyst was still intact after 48 h of reaction (Figure S12). The photocatalyst can also be recycled from a 24 h reaction mixture with a separation yield of 95% (separation details can be found in the Experimental Section), and the recycled photocatalyst can still give a 34% yield of benzoic acid after a second 24 h reaction (Figure S13).

Table 1 shows the control experiments for PS deconstruction and the optimizations of the photocatalysts. No reactivity was observed without the photocatalyst (entry 2) or light (entry 8, temperature still at 50 °C), proving that the polymer deconstruction is a photocatalytic (instead of thermal catalytic) process. While 20 mol % loading of the catalyst is required to produce benzoic acid with 38 ± 3% yield at 48 h (entry 1), the reaction with 5 mol % of fluorenone can still achieve 31% of benzoic acid at 48 h (entry 3), further confirming the catalytic role of fluorenone. The control experiment without the PS substrate did not deliver any product (entry 4), showing that the aromatic product was generated from PS instead of fluorenone. No reactivity was observed without the acid (entry 5), whereas the use of other acids (e.g., HNO_3_, H_3_PO_4_, HClO_4_, and CF_3_SO_3_H) can still produce benzoic acid (Table S1). The specific role of the acid is discussed in the mechanistic study section below. A lower loading of H_2_SO_4_ (0.25 equiv, entry 6) can still give a reasonable yield of benzoic acid (22%), confirming the feasibility of this reaction with a more diluted acid.

**Table 1:**
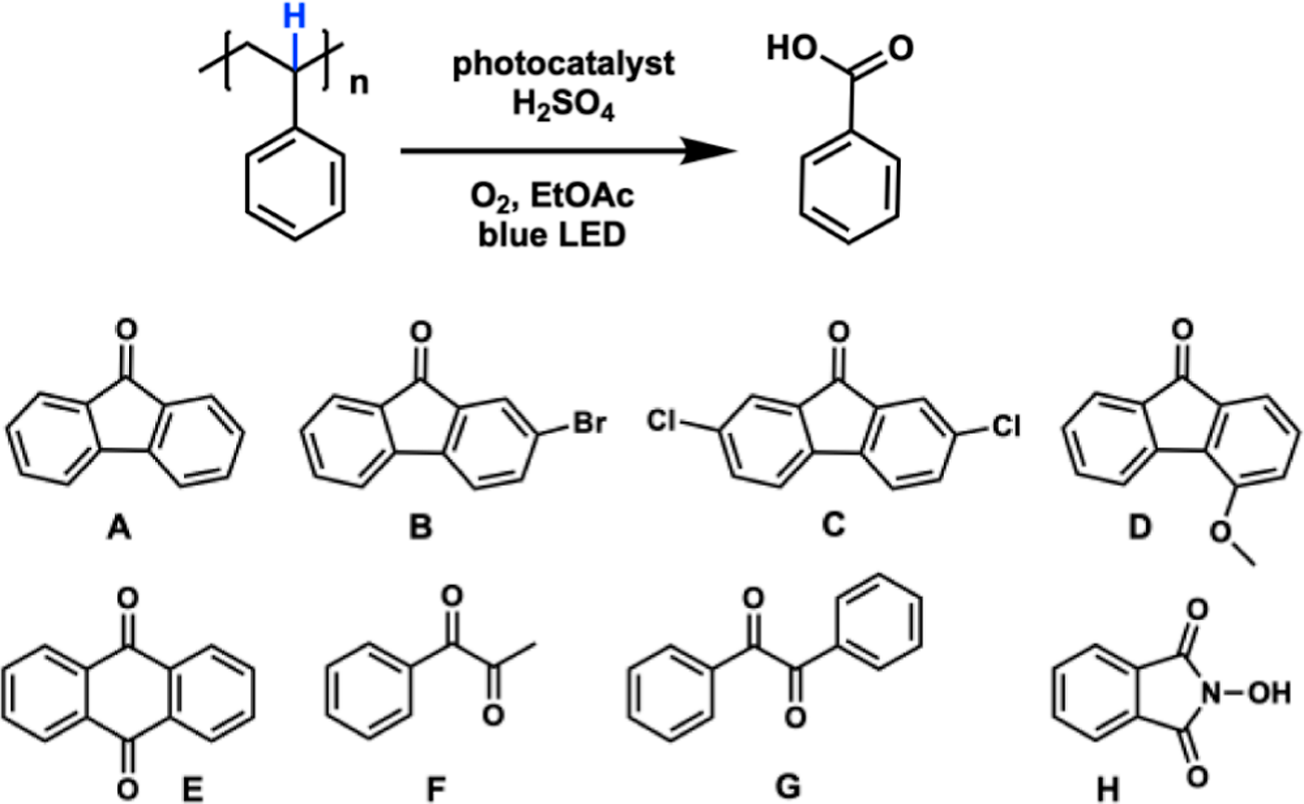
Control Experiments for PS Deconstruction and Optimization of Photocatalysts

entry	PS	catalyst	H_2_SO_4_	oxygen source	light	benzoic acid yield^a^
1	0.1 M	**A** (20 mol %)	1 equiv	O_2_ balloon	blue LED	30 ± 2% (38 ± 3%)^b^
2	0.1 M	no catalyst	1 equiv	O_2_ balloon	blue LED	0%
3	0.1 M	**A** (5 mol %)	1 equiv	O_2_ balloon	blue LED	21% (31%)^c^
4	no PS	**A** (20 mol %)	1 equiv	O_2_ balloon	blue LED	0%
5	0.1 M	**A** (20 mol %)	0 equiv	O_2_ balloon	blue LED	0%
6	0.1 M	**A** (20 mol %)	0.25 equiv	O_2_ balloon	blue LED	22%
7	0.1 M	**A** (20 mol %)	1 equiv	N_2_ balloon	blue LED	0%
8	0.1 M	**A** (20 mol %)	1 equiv	O_2_ balloon	dark, 50 °C	0%
9	0.1 M	**B** (20 mol %)	1 equiv	O_2_ balloon	blue LED	28%
10	0.1 M	**C** (20 mol %)	1 equiv	O_2_ balloon	blue LED	22%
11	0.1 M	**D** (20 mol %)	1 equiv	O_2_ balloon	blue LED	16%
12	0.1 M	**E** (20 mol %)	1 equiv	O_2_ balloon	blue LED	12%
13	0.1 M	**F** (20 mol %)	1 equiv	O_2_ balloon	blue LED	21%
14	0.1 M	**G** (20 mol %)	1 equiv	O_2_ balloon	blue LED	29%
15	0.1 M	**H** (20 mol %)	0 equiv	O_2_ balloon	blue LED	5.5%^d^

a 2 mL EtOAc, 16 h reaction.

b 30 ± 2% yield at 16 h, 38 ± 3% yield at 48 h.

c 21% yield at 16 h, 31% yield at 48 h.

d Carbon nitride (10 mg) was used together with **H**.

A series of photocatalysts were studied for the conversion of PS to benzoic acid (Table 1, entries 9–15; full optimizations of photocatalysts can be found in Figure S14). A wide range of compounds are demonstrated to be capable of facilitating this reaction. Fluorenone (A) performed as the most efficient photocatalyst, with compounds B, C, F, and G showing similar yields. It is also notable that 5.5% benzoic acid was produced when compound H (*N*-hydroxyphthalimide, NHPI) was used in combination with carbon nitride (a heterogeneous light absorber, see the Supporting Information). No reactivity was observed when NHPI was used on its own because it cannot absorb visible light. NHPI is known as a common HAT redox mediator for C–H oxidation,^20,27^ which has a different catalytic structure (N-oxyl) from that of the aromatic ketone catalysts, and therefore, the successful PS deconstruction catalyzed by NHPI provides further evidence for the HAT route for oxidative polymer deconstruction. More optimizations of PS concentration, solvent, acid, oxygen source, and light source are shown in Table S1. The final optimized reaction conditions are as follows: 0.1 M PS, 0.2 equiv of fluorenone, 1 equiv of H_2_SO_4_, O_2_ balloon, and blue LED.

### Gram-Scale Deconstruction of Pure PS and Real-World PS Foam

**Figure 3:**
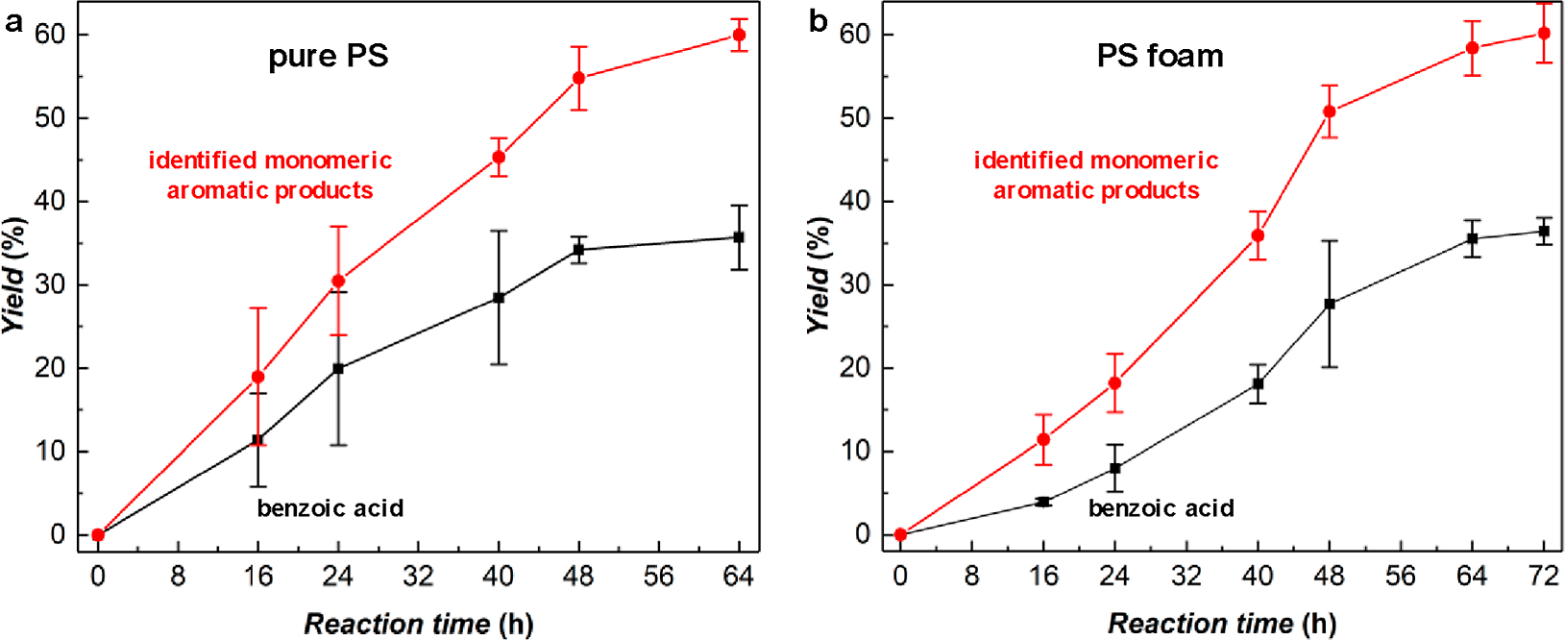
Gram-scale deconstruction for pure PS and real-world PS foam. Yield of all identified aromatic products (red) and yield of benzoic acid (black) over the reaction time for the deconstruction of (a) pure PS and (b) real-world PS foam. Reaction conditions: 10 mmol (1.04 g) PS, 0.2 equiv of fluorenone, 1 equiv of H_2_SO_4_, 20 mL of EtOAc, O_2_ balloon, blue LED, 50 ± 3 °C (due to LED irradiation). Error bars correspond to the standard deviation of triplicate experiments. The remaining ∼40% yield of aromatic products at 48 h are aromatic oligomers.

We next performed a gram-scale (10 mmol, 1.04 g) deconstruction of PS (Figure 3a), where the yield of all identified aromatic products was 60 ± 2% (including 36 ± 4% of benzoic acid) at 64 h. The relatively large errors at the early stage of the reactions suggested that the initial reaction rates are sensitive to the experimental conditions (e.g., light intensity of LED, temperature, etc.). However, the error bars became smaller as the reactions proceeded, indicating that the experiments appearing slower at the beginning gave similar yields at the end. A gram-scale reaction for real-world PS foam (expanded PS packaging foam; *M*_w_ ∼ 250 000, PDI ∼ 4.2) was also carried out (Figure 3b, Movie S1), which delivered aromatic products with 60 ± 4% yield (including 36 ± 2% of benzoic acid) at 72 h. Benzoic acid was isolated with yields of 40% and 38% for the gram-scale reaction of pure PS and PS foam, respectively (^1^H NMR for the isolated benzoic acid shown as Figure S15). These results demonstrated the practicality and potential scalability of this polymer deconstruction reaction.

### Mechanistic Study

**Figure 4:**
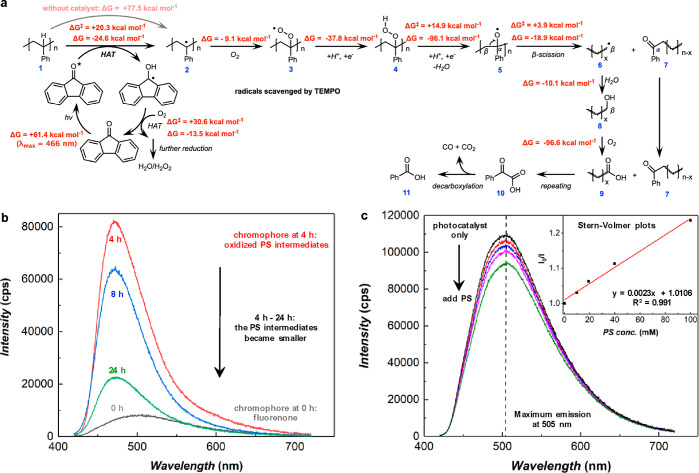
Mechanistic study. (a) Proposed reaction mechanism. Free energy changes (Δ*G*) and kinetic energy barrier (Δ*G*^‡^) were calculated by DFT with a styrene dimer model compound. (b) Fluorescence spectra for PS deconstruction reaction mixtures at 0 h (black), 4 h (red), 8 h (blue), and 24 h (green). (c) Fluorescence spectra of the photocatalyst before and after adding PS into the solution. Inset: Stern–Volmer plots. Fluorescence quenching experiments were performed by adding different concentrations of PS (10–100 mM in EtOAc) into the fluorenone photocatalyst solution (10 mM in EtOAc) under a N_2_ atmosphere. The PS solution also contained a 10 mM photocatalyst to maintain a constant concentration of the photocatalyst in each fluorescence measurement. The fluorescence emission at 505 nm was used for Stern–Volmer plots as follows: *I*_0_/*I* = 1+ *k*_q_τ_0_[*Q*]. *I*_0_: fluorescence intensity for the photocatalyst only (without PS); *I*: fluorescence intensity measured with PS; *k*_q_: rate constant of quenching; τ_0_: lifetime of the excited state of the photocatalyst without a quencher; [*Q*]: concentration of the quencher (PS). Values of *I*_0_/*I* are taken from the average of triplicate experiments. Excitation wavelength for fluorescence was set at 405 nm.

The reaction mechanism was investigated for PS deconstruction (Figure 4a), and the energy change for each step was calculated by density functional theory (DFT) with a styrene dimer model compound. Details of DFT calculations can be found in the Experimental Section. The activation of the fluorenone photocatalyst requires an energy input of 61.4 kcal mol^–1^ (energy diagram shown as Figure S16), which is provided by blue LED (corresponding λ_max_ = 466 nm, consistent with the LED emission spectrum). The HAT process between PS (compound **1**) and the excited state of the photocatalyst produces PS radicals (**2**, stabilized by the aromatic structure) and the ketyl radical, followed by reduction of O_2_ to regenerate the photocatalyst (Figures S17 and S18).^24^ The O_2_ is reduced to H_2_O_2_ and/or H_2_O (detection of H_2_O_2_ is shown in Figure S19). The free energy change (Δ*G*) and kinetic energy barrier (Δ*G*^‡^) for the fluorenone-catalyzed HAT step are −24.6 and +20.3 kcal mol^–1^, respectively. In contrast, the Δ*G* for the HAT step without the photocatalyst is +77.5 kcal mol^–1^, confirming the catalytic role of fluorenone. The PS radicals are subsequently oxygenated to form compound **3** (Δ*G* = −9.1 kcal mol^–1^, energy diagram shown as Figure S20),^12^ followed by HAT of the peroxyl radical group to give **4** (Δ*G* = −37.8 kcal mol^–1^) and removal of H_2_O to give **5** (Δ*G* = −96.1 kcal mol^–1^, Δ*G*^‡^ = +14.9 kcal mol^–1^, Figure S21).

Next, the reaction proceeds through a β-scission process (Δ*G* = −18.9 kcal mol^–1^, Δ*G*^‡^ = +3.9 kcal mol^–1^, Figure S21) involving the cleavage of the C_α_–C_β_ bond to generate the radical species (**6**) and the terminal aromatic C=O group (**7**). This β-scission mechanism is in alignment with previous reports.^36,37^ The C–C bond cleavage without H^+^ participation has a much larger Δ*G*^‡^ (+66.1 kcal mol^–1^, Figure S22), explaining the key role of the acid. A stoichiometric amount of acid is required in the removal of H_2_O from **4** and the oxygen reduction reaction (i.e., the sacrificial reaction). Compound **6** will be oxidized and hydrated to form alcohol **8** (Δ*G* = −10.1 kcal mol^–1^), which will be further oxidized to form a carboxylic acid (**9**, Δ*G* = −96.6 kcal mol^–1^).^35^ Continuous breakdown by the aforementioned processes will generate phenylglyoxylic acid (**10**), which can undergo decarboxylation to form benzoic acid (**11**) and CO_2_/CO. An alternative pathway (retro-aldol reaction) for breaking the C–C bond is shown in Figure S23, which can explain the formation of the small amount of acetophenone byproduct. The ethyl benzoate byproduct can be generated from the reaction of **10** or **11** with the ethyl acetate solvent.

A series of experiments were performed to support the proposed mechanism. First, the phenylglyoxylic acid intermediate (**10**) was detected by GC–MS, and a control experiment with **10** also confirmed its photoconversion to benzoic acid under the experimental conditions of this work (Figure S24). Second, adding a radical scavenger such as TEMPO stopped the polymer deconstruction process, suggesting that radicals are key intermediates formed in the reaction. Third, a significant change of fluorescence emission was observed during the PS deconstruction (Figure 4b). At 0 h, the fluorescence was generated by the fluorenone photocatalyst. An increase of fluorescence intensity and a blue shift of the maximum emission wavelength were observed after 4 h of reaction, indicating the formation of PS intermediates containing conjugated C=O groups (**7**), which can act as a chromophore that significantly enhances the fluorescence in the reaction mixture. As the degradation reaction carried on, the oxidized PS intermediates became smaller (i.e., the size of the conjugated system decreased), and therefore, the fluorescence decreased. A similar trend for light absorption was also observed by UV–vis spectrometry (Figure S25).

In order to study the electron transfer between the excited state of the photocatalyst and the polymer substrate, Stern–Volmer fluorescence quenching experiments were also performed (Figure 4c). Fluorescence emission of the fluorenone photocatalyst (10 mM in EtOAc) was first measured, which exhibited a maximum intensity at 505 nm (*I*_0_). The PS solution (also containing a 10 mM photocatalyst to maintain a constant concentration of the photocatalyst) was then added into the photocatalyst solution (PS did not show any light absorption or fluorescence in this wavelength range, Figure S26). The fluorescence emission intensity (*I*) was significantly decreased as more PS was added. The inset in Figure 4c shows a linear relationship between *I*_0_/*I* and PS concentration, suggesting that the excited state of the photocatalyst was quenched by PS. The Stern–Volmer plots exhibit a slope of 2.3 M^–1^, which reflects the rate of quenching and is similar to the slope value in a previous report on fluorenone-catalyzed small-molecule reactions (1.3 M^–1^).^35^ These results demonstrated that the HAT process between a photocatalyst and a polymer substrate can occur in a similar way to small-molecule catalysis and provided support for the proposed mechanism.

## Conclusions

The deconstruction of PS to produce benzoic acid was demonstrated under photocatalytic conditions and the reaction is proposed to proceed via a C–H oxidation pathway. The catalytic system is scalable and can produce useful organic feedstocks from real-world plastic waste. This work shows the deconstruction of addition polymers that (i) delivers a product that is more valuable than CO_2_; (ii) is performed under relatively mild conditions (ambient temperature and pressure, aerobic oxidation, driven by visible light); and (iii) does not require an expensive or toxic catalyst. This work also bridges the gap between polymer deconstruction and organic catalysis and encourages the polymer degradation community to design new depolymerization reactions based on existing small-molecule catalysis techniques. Furthermore, this strategy can potentially be applied to other addition polymers, such as PP, PVC, and PE, although a different HAT photocatalyst with stronger oxidation ability than fluorenone may be required to oxidize the polymers that are more inert than PS. In order to drive upscaled plastic deconstruction that is more relevant to industrial implementation, issues related to product separation, substrate replenishment, and catalyst recycling still need to be improved, which could potentially be addressed by a continuous flow reactor in the future. The use of diluted H_2_SO_4_ is currently a drawback for plastic deconstruction in this work, but it can potentially be mitigated by using H_2_SO_4_ sourced from waste feeds.^38,39^ This work highlights an opportunity for plastic recycling industries to convert plastic waste into valuable chemicals and helps to address the serious environment problem of plastic pollution.

## Experimental Section

### Materials

Ethyl acetate (chemically pure), tetrahydrofuran (HPLC grade), 9-fluorenone (99%), H_2_SO_4_ (95%), and PS (*M*_w_ ∼ 260 000; *M*_n_ ∼ 105 000) were purchased from commercial vendors (Alfa Aesar, Fisher Scientific, Sigma-Aldrich, or ACROS Organics) and used as received. The blue LED strip (14.4 W, 1 m) was purchased from LEDXON MODULAR and shaped into a roll (diameter ∼ 7 cm, height ∼ 5 cm). Real-world expanded PS (*M*_w_ ∼ 250 000; *M*_n_ ∼ 59 000) packaging foam was cut into smaller pieces and dissolved in solvents without additional treatment.

### Photochemistry

The polymer solution was prepared by dissolving 0.208 g of PS (corresponding to 2 mmol styrene monomer) in 20 mL of EtOAc and sonicating for ∼5 min to make a homogeneous solution. H_2_SO_4_ (2 mmol, 1 equiv) and fluorenone (0.4 mmol, 0.2 equiv) were then added to the solution. For each experiment, 2 mL of solution (containing 0.2 mmol PS) was added into a 10 mL borosilicate vial, and two vials were placed in one LED roll (14.4 W). The blue LED created a local temperature of around 50 ± 3 °C for the reaction (measured by a thermometer). The vial was sealed by a septum, and oxygen was provided by a balloon filled with pure O_2_ gas (balloon diameter ∼25 cm). A photochemical experiment was performed under blue LED irradiation with magnetic stirring.

For the upscaled reaction, the polymer solution was prepared by dissolving 10 mmol of PS chemical reagent or real-world PS into 20 mL of EtOAc and sonicating for ∼30 min to make a homogeneous solution. H_2_SO_4_ (10 mmol, 1 equiv) and fluorenone (2 mmol, 0.2 equiv) were added to the solution. The addition of H_2_SO_4_ may lead to precipitation of the polymer, which requires sonication until the solution is clear again. For each experiment, 20 mL of solution was added into a 50 mL Schlenk tube, and each Schlenk tube was individually placed in an LED roll. The tube was sealed by a septum, and oxygen was provided by two O_2_ balloons. The balloons were refilled every 24 h to ensure that the reaction was provided with sufficient O_2_. The photochemical experiment was performed under blue LED irradiation with magnetic stirring.

Photoconversion of PS (0.2 mmol) was also performed with cyanamide-functionalized carbon nitride (NCN-CN_*x*_, 5.0 mg) and NHPI (0.2 equiv) in a similar way as described above, except that no H_2_SO_4_ was used. The NCN-CN_*x*_ was synthesized according to reported methods,^40^ where pristine CN_*x*_ was first formed by heating melamine at 550 °C, followed by functionalization with potassium thiocyanate at 400 °C for 1 h and 500 °C for 30 min.

### Product Analysis

The reaction mixture of PS deconstruction was analyzed by a Waters high-performance liquid chromatograph equipped with a Waters Acquity C18 column (2.1 × 50 mm), a SQD2 single quadrupole mass-spec detector, and a diode array UV–vis detector. The peak of benzoic acid was identified by confirming the molecular weight of benzoic acid (*M*_w_ = 122) by a mass spectrometry detector as well as comparing with a standard sample of benzoic acid. For quantitative analysis, a series of standard solutions with known concentrations of benzoic acid were prepared, and 1-tetralone was added as an internal standard. The UV–vis absorption peak of benzoic acid was normalized to the internal standard peak to plot a linear calibration curve. For each HPLC measurement, 50 μL of the reaction solution was diluted with 950 μL of EtOAc so that the concentration of benzoic acid was kept below 4 mM.

The reaction mixture was also analyzed by ^1^H NMR spectroscopy to confirm the formation of benzoic acid. The peaks of benzoic acid were identified by observation of an increase of the peaks after adding an authentic sample of benzoic acid into the sample. The NMR data were collected using a Bruker Neo Prodigy 400 MHz NMR spectrometer. For each NMR measurement, 250 μL of deuterated dichloromethane (CD_2_Cl_2_) was added into 500 μL of the reaction solution.

The molecular weight of the polymer was analyzed by GPC. The reaction mixture was first evaporated to remove EtOAc, followed by dissolution in chlorobenzene and analysis by an Agilent Technologies 1200 series gel permeation chromatograph equipped with a refractive index detector. The reaction mixtures were also analyzed by gas chromatography–mass spectrometry (GC–MS) with a Shimadzu QP2010-SE gas chromatograph fitted with a SHIM-5MS column (30 m, 0.25 mm, 0.25 μm film). GC–MS peaks were identified using the mass spectra of the peaks with compounds in the NIST Mass Spectrometry Data Center database (probability match > 90%). The UV–vis spectra of the reaction mixture were collected using an Agilent Cary 60 UV–vis spectrometer. The fluorescence spectra were recorded on an Edinburgh Instruments FS5 spectrofluorometer. The excitation wavelength was set at 405 nm, and the emission spectroscopy was collected.

The gaseous products were analyzed by injecting 50 μL of gas from the headspace of the tube into the gas chromatograph. CO was quantified with a Shimadzu Tracera 2010 gas chromatograph equipped with a barrier discharge ionization detector and a RT-Molsieve 5A (30 m × 0.53 mm ID, Restek) column. He (BOC, UK) was used as a carrier gas. CO_2_ was quantified using an Agilent 7890A gas chromatograph equipped with a thermal conductivity detector. A HP Plot Q (30 m × 0.53 mm ID, Agilent Technologies) column was used to separate CO_2_ from the gas aliquot, and it was detected by the thermal conductivity detector. N_2_ (BOC, UK) was used as a carrier gas. The gas in the balloon was analyzed separately in a similar way. The total amount of gas product was calculated by combining the amount of gas product in the headspace and the balloon. Calibration curves for CO and CO_2_ were created by plotting the peak area versus concentration in standard calibration gas.

### Product Separation and Photocatalyst Recycling

After the gram-scale PS deconstruction, water (10 mL) was added to the reaction mixture, and the organic layer was extracted with CH_2_Cl_2_ (20 × 3 mL), washed with brine (10 mL), dried over Na_2_SO_4_, filtered, and concentrated in vacuo. The crude product was applied to flash chromatography on silica gel, and the pure benzoic acid was isolated in a 10% ethyl acetate/petrol ether solvent system. For the photocatalyst recycling experiment, water (4 mL) was added to the reaction mixture, and the organic layer was extracted with CH_2_Cl_2_ (10 × 3 mL), washed with brine (4 mL), dried over Na_2_SO_4_, and concentrated in vacuo. The crude product was applied to flash chromatography on silica gel, and fluorenone was isolated in a 2% ethyl acetate/petroleum ether solvent system. The isolated fluorenone was then used as a photocatalyst for the second run of the reaction.

### Fluorescence Quenching Experiment

All sample preparations and measurements were performed under N_2_. The photocatalyst solution (10 mM in EtOAc) was first measured without the polymer substrate, and the fluorescence intensity at 505 nm was set as *I*_0_. The PS solution (0.5 M in EtOAc) containing the 10 mM photocatalyst (in order to maintain a constant concentration of photocatalyst) was added into the photocatalyst solution. The volume of the PS solution added was adjusted to give a PS concentration of 10, 20, 40, and 100 mM for fluorescence quenching measurements. The fluorescence intensity after adding PS was recorded as *I*. *I*_0_/*I* was plotted against the concentration of PS (Stern–Volmer plot) to show that the fluorescence of the photocatalyst was quenched by the PS substrate. Saturation with O_2_ did not change the fluorescence intensity.

### DFT Calculations

In order to minimize the computational cost associated with modeling the photoconversion process of PS, all DFT calculations were performed using a “distyrene” unit of the polymer chain. All calculations were carried out using the Gaussian16 software package.^41^ First, all geometry optimizations were performed using the B3LYP hybrid exchange–correlation functional together with the standard 6-31G* basis set for all atoms.^42−44^ An extra quadratic convergent self-consistent field (SCF) step was added when the first-order SCF did not converge (“scf = xqc” keyword). Solvent effects (ethyl acetate) have been considered in the calculations with the polarizable continuum model-solvation model based on density (PCM-SMD) of Truhlar and co-workers.^45−47^ Dispersion effects were also included using the Grimme D3 correction.^48^ Then, the located stationary points were characterized by analytical harmonic frequency calculations at the same level of theory as the geometry optimizations. Additionally, the energy of the geometry-optimized molecules was refined by a single-point calculation with the cc-pVTZ basis set for all atoms.^49,50^ The dispersion- and solvent-corrected time-dependent DFT excitation energy of fluorenone was calculated using the M062x density functional together with the Def2TZVP basis set as this combination yielded results that correlated the best with the experimental data. Gibbs energy values (G) were obtained by including thermal corrections to the potential energy computed with the cc-pVTZ basis set on equilibrium geometries. Intrinsic reaction coordinate calculations have been performed to verify the connectivity between the transition states and the found reagent and product minima structures.^51,52^ All relative energy values discussed and displayed throughout this article are in kcal mol^−1^ and were calculated at 298 K unless otherwise specified.

## Supporting Information Available

PS foam dissolving in ethyl acetate (MOV)
Emission spectrum of the blue LED light; experimental setup; ^1^H NMR, HPLC, GC–MS, LC–MS, GPC, and UV–vis spectra for the reaction mixture; detection of gas products; temporal changes and reusability of the fluorenone photocatalyst; list of different photocatalysts; ^1^H NMR spectra for isolated benzoic acid; calculated energy diagrams for each step of the reaction; detection of H_2_O_2_; alternative reaction pathway for C–C bond cleavage; control experiment of phenylglyoxylic acid; UV–vis and fluorescence spectra for PS and fluorenone; optimization of the reaction conditions; and calculated cartesian coordinates for intermediates (PDF)

## Supplementary Material

cs2c02292_si_001.mov
