# Supplementary material for: Bridging Plastic Recycling and Organic Catalysis: Photocatalytic Deconstruction of Polystyrene via a C–H Oxidation Pathway
Source: ACS Catal. 2022 Jun 23;12(14):8155–63. doi: 10.1021/acscatal.2c02292 (PMC9295126; doi:10.1021/acscatal.2c02292)
Supplement: Supplementary file 2 — cs2c02292_si_002.pdf [file cs2c02292_si_002.pdf]

Supporting Information

Bridging Plastic Recycling and Organic Catalysis: Photocatalytic Deconstruction of Polystyrene via C-H Oxidation Pathway

Tengfei Li[1,2], Arjun Vijeta[1], Carla Casadevall[1], Alexander Gentleman[3], Tijmen Euser[3], Erwin Reisner[1]*

[1]Yusuf Hamied Department of Chemistry, University of Cambridge, Cambridge CB2 1EW, UK
[2]Department of Natural Sciences, Manchester Metropolitan University, Manchester M1 5GD, UK
[3]Cavendish Laboratory, University of Cambridge, Cambridge, CB3 0HE, UK
*Correspondence to: reisner@ch.cam.ac.uk

$$n_{polystyrene}(mol) = \frac{weight\ of\ polystyene\ (g)}{M.W_{\cdot styrene\ monomer}\ \ (104\ g\ mol^{-1})} \qquad Eq.S1$$

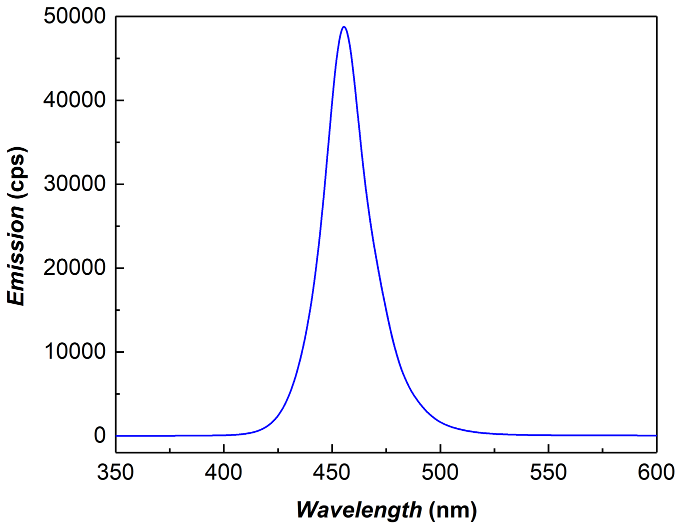

Figure S1. Emission spectrum for the blue LED light source.

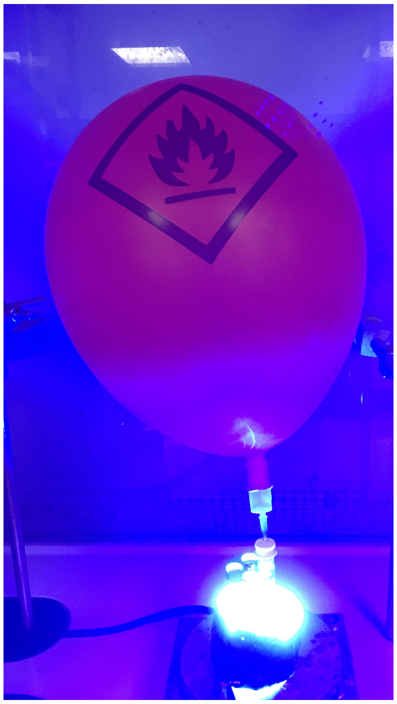

Figure S2. Experimental setup. Polymer solution (2 mL) containing fluorenone and H_2SO_4 was kept in a sealed borosilicate vial (~10 mL) and irradiated by a blue LED. O_2 balloon (diameter ~25 cm) was connected through a needle.

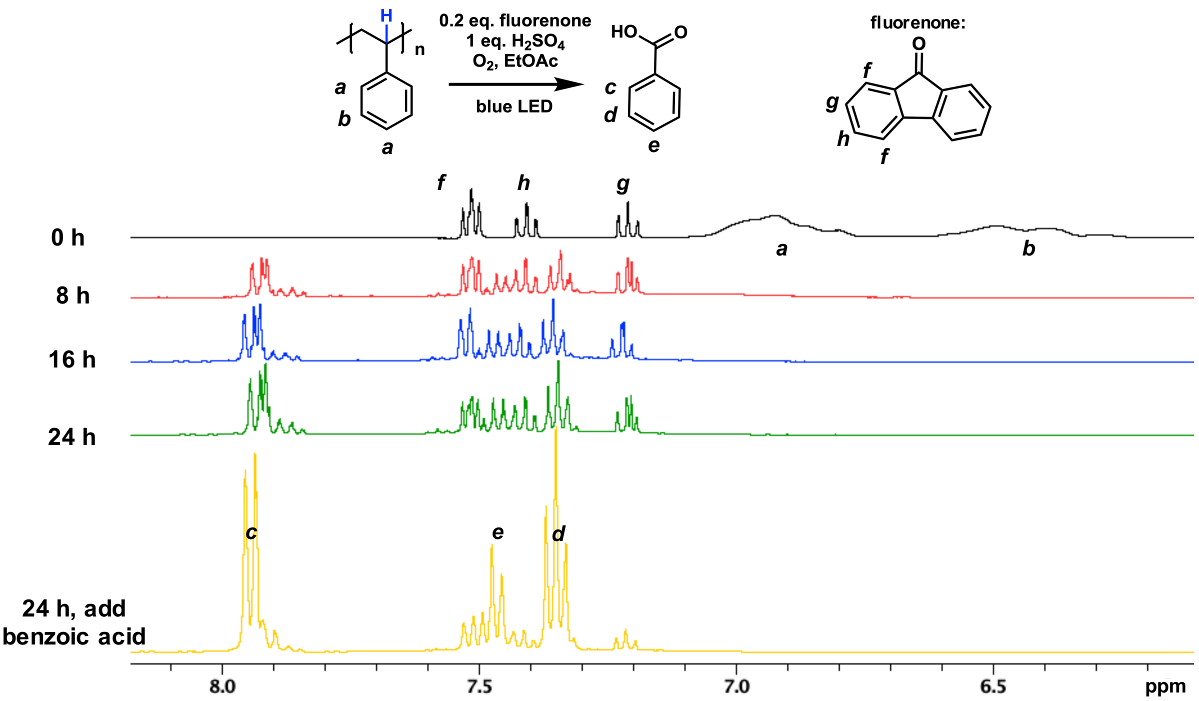

Figure S3. Crude ^{1}H NMR spectra (128 scans) for the reaction mixtures at 0 h, 8 h, 16 h, 24 h. A significant increase of the benzoic acid peaks was observed after adding benzoic acid into the 24-h sample. CD_2Cl_2 was added as the deuterated reagent for the ^{1}H NMR measurement. Reaction conditions: 0.1 M PS, 0.2 equiv. fluorenone, 1 equiv. H_2SO_4, 2 mL EtOAc, O_2 balloon, blue LED irradiation, 50 ± 3 °C. The aromatic peaks of fluorenone, PS and benzoic acid are labelled: polystyrene (a and b); benzoic acid (c, d and e); fluorenone (f, g and h).

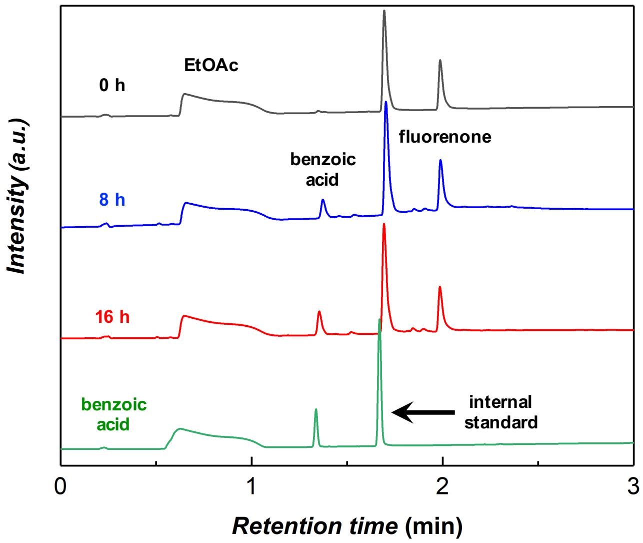

Figure S4. HPLC traces (UV-Vis detector) for the reaction mixtures at 0 h, 8 h, 16 h, and standard benzoic acid. Internal standard (1-tetralone) was added for quantification of the product. Reaction conditions: 0.1 M PS, 0.2 equiv. fluorenone, 1 equiv. H_2SO_4, 2 mL EtOAc, O_2 balloon, blue LED irradiation, 50 ± 3 °C.

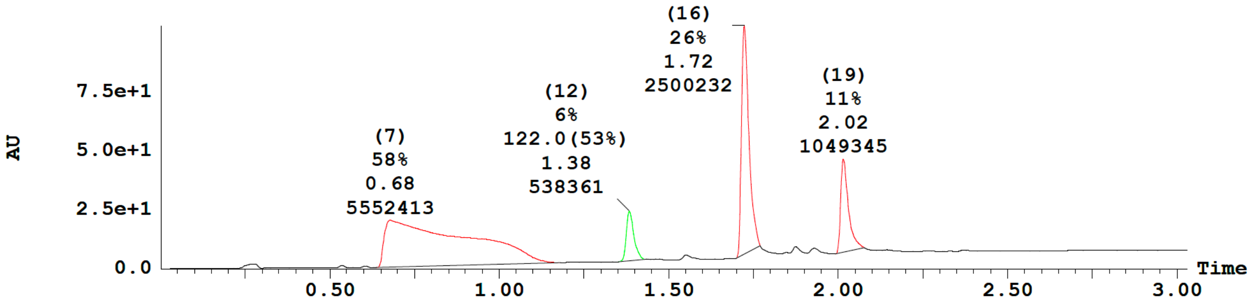

Peak Number	Compound	Time	AreaAbs	Area %Total	Width	Height	Mass Found
7		0.68	6e+006	57.60	1	2e+007	Not Found
12	Found	1.38	5e+005	5.58	0	2e+007	Found, 122.00
16		1.72	3e+006	25.94	0	1e+008	Not Found
19		2.02	1e+006	10.88	0	4e+007	Not Found

Molecular weight of benzoic acid: 122

Figure S5. HPLC result (UV-Vis detector, with mass-spec detector to analyze the molecular weight of each separated compound) for PS deconstruction at 16 h. Benzoic acid (molecular weight = 122) was detected and highlighted. Reaction conditions: 0.1 M PS, 0.2 equiv. fluorenone, 1 equiv. H_2SO_4, 2 mL EtOAc, O_2 balloon, blue LED irradiation, 50 ± 3 °C.

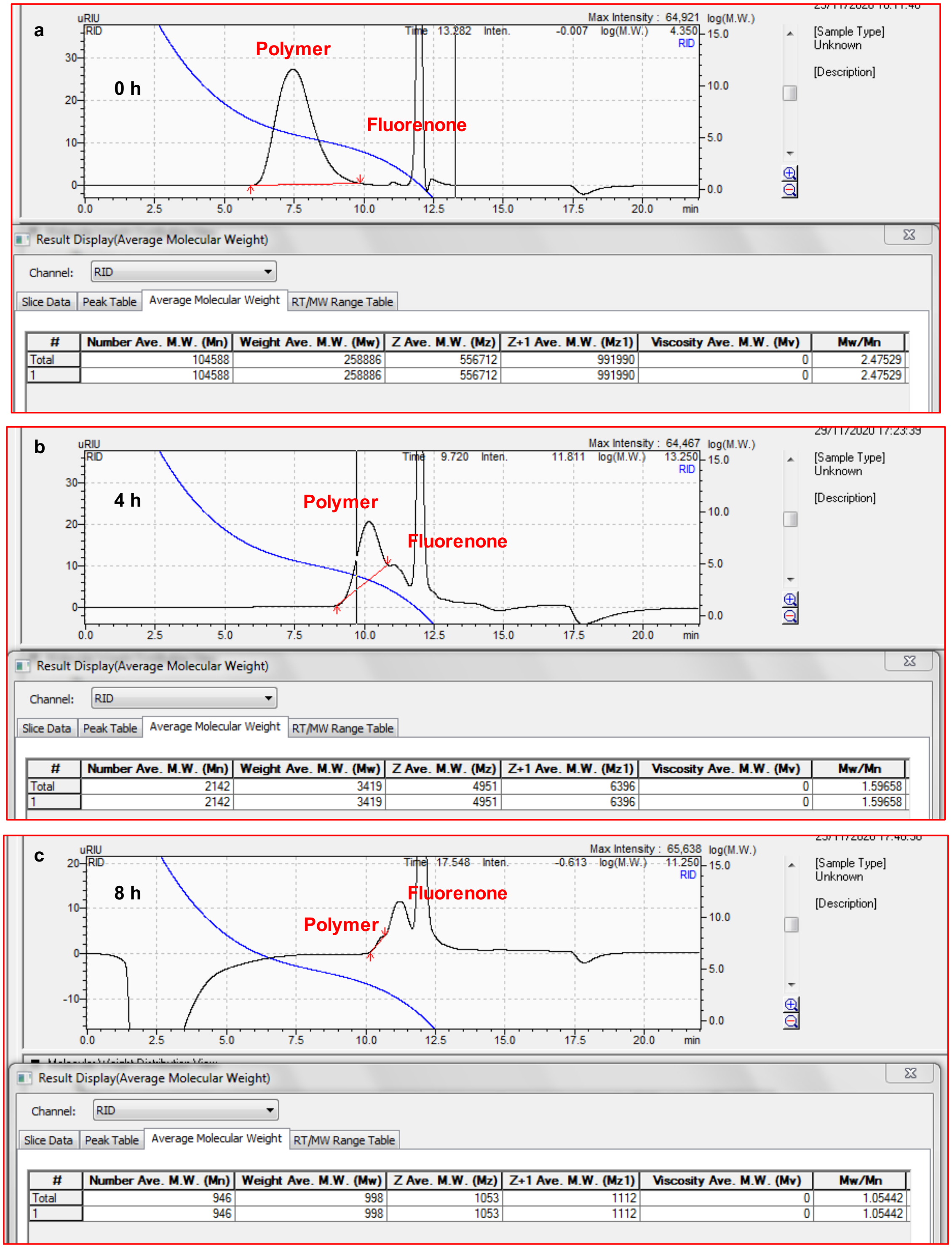

Figure S6. GPC results for PS deconstruction at 0 h (a), 4 h (b) and 8 h (c). The peaks of polymer are highlighted and the molecular weight information is provided, including number average molecular weight (M_n), weight average molecular weight (M_w) and PDI (equals to M_w/M_n). The increase of the retention time for the polymer peak correspond to the decrease of molecular weight. Signals were recorded by a refractive index detector (RID). The peak at 12.4 min is attributed to the fluorenone catalyst.

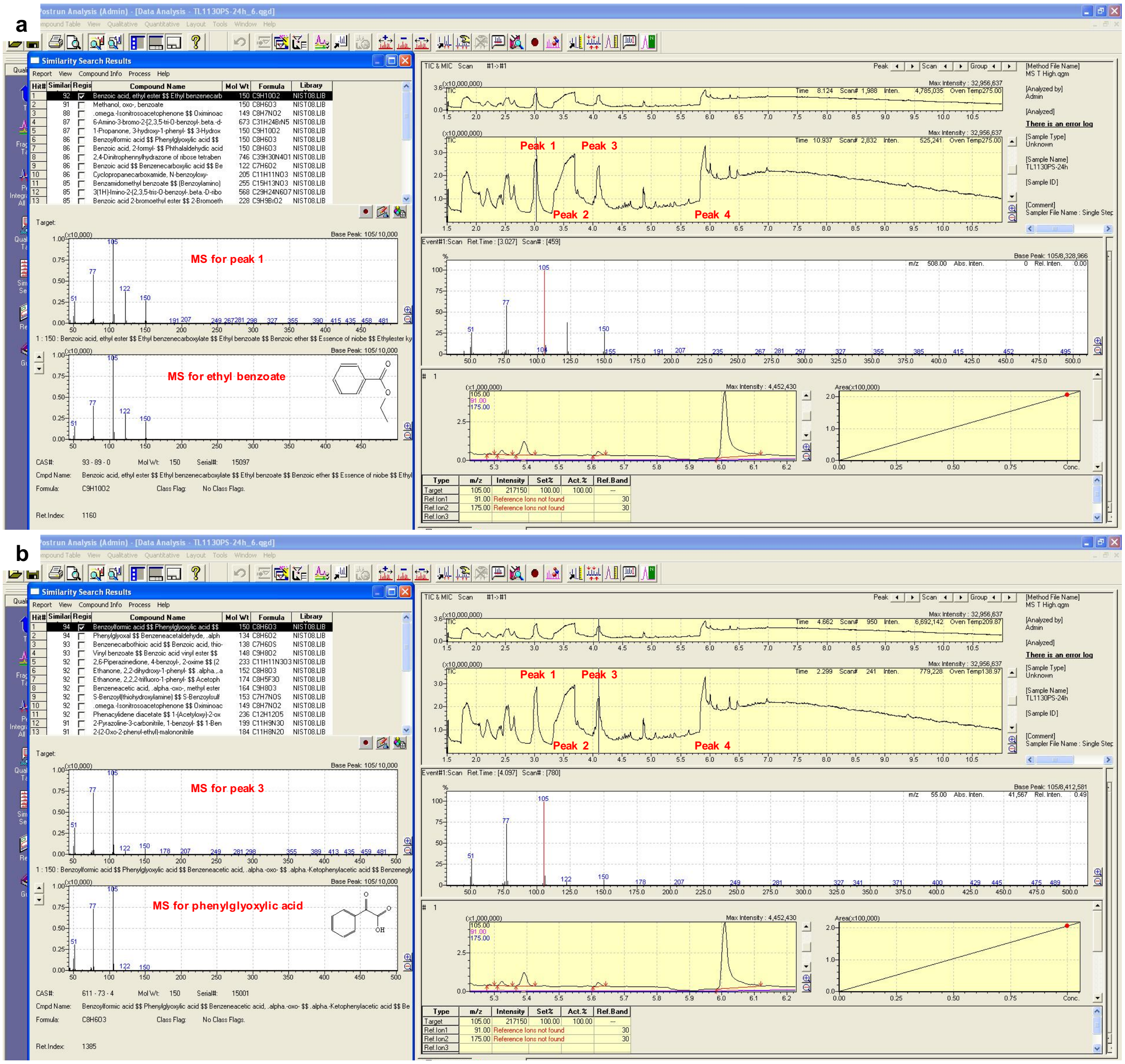

Figure S7. GC-MS spectra for the reaction mixture at 24 h. Peak 1 (a) and peak 3 (b) were identified as ethyl benzoate and phenylglyoxylic acid, respectively, by comparing the mass spectra of the peaks with compounds in the NIST Mass Spectrometry Data Center database (probability match >90%). Peak 1 and peak 4 were identified as benzoic acid and fluorenone, respectively. The peaks before 3 min were silica impurities from the cap of the sample vial.

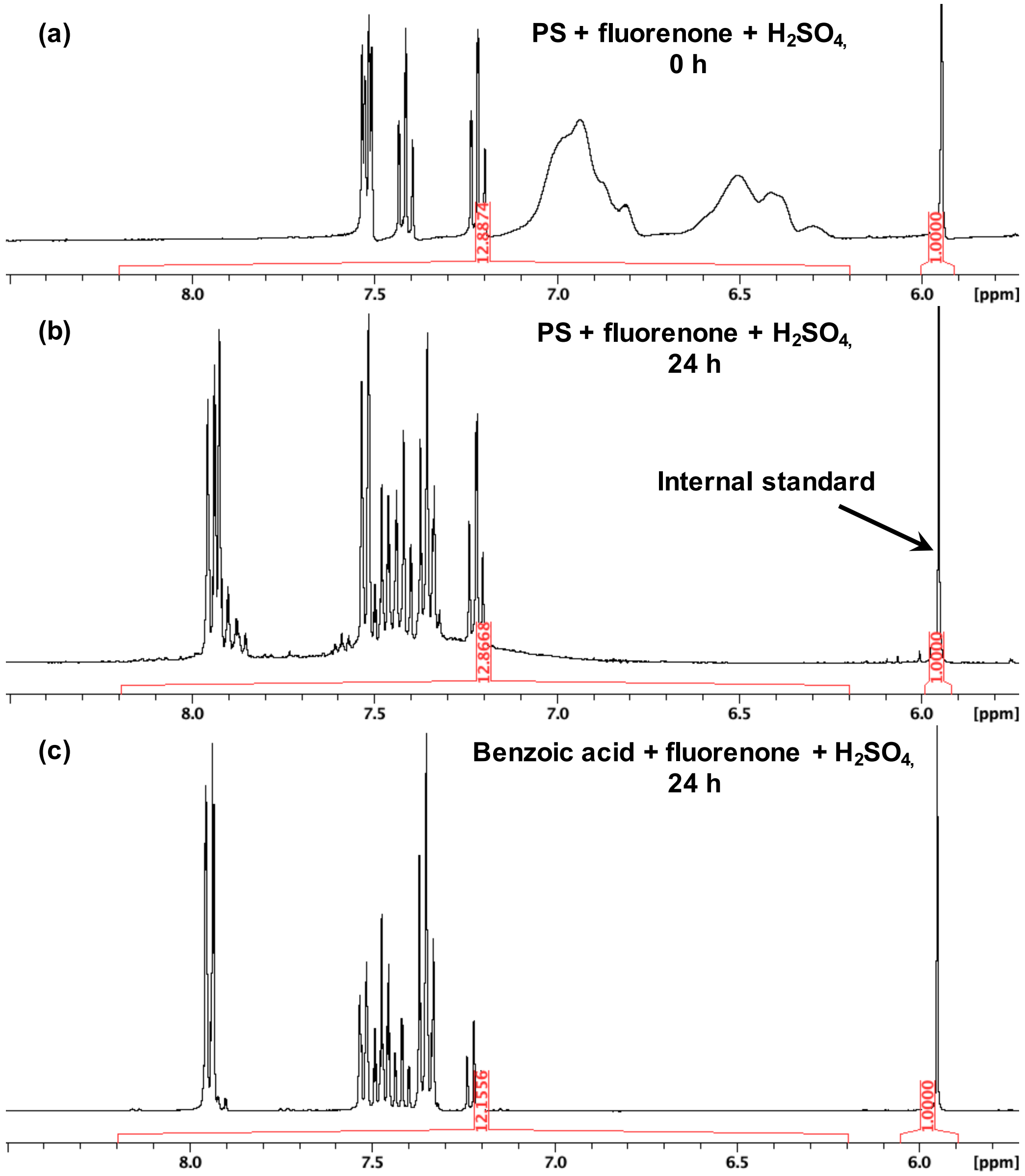

Figure S8. ^{1}H NMR spectra and the integration results of the aromatic region (6.2 ppm to 8.2 ppm) that show mass balance of the reaction. 1,3.5-trimethoxybenzene was used as the internal standard, and the area of its singlet peak at 6.95 ppm was set as 1 for integration. Reaction conditions: 0.1 M PS (for a and b) or 0.1 M benzoic acid (for c); 0.02 M fluorenone; 0.1 M H_2SO_4; blue LED for 24 h. (a) NMR spectra measured before PS deconstruction reaction. Integration of the aromatic region was 12.89. (b) NMR spectra measured after 24 h PS deconstruction reaction. Integration of the aromatic region was 12.87, which showed 100% mass balance. (c) NMR spectra measured after 24 h photocatalytic reaction at the same conditions as (b), except that PS was replaced by benzoic acid. No conversion of benzoic acid was observed. Integration of the aromatic region was similar to (a) and (b). Note that the bump (7.0 ppm to 7.7 ppm) in (b) was consistently observed and not due to instrument baseline problem or the use of acid, because the control experiment in (c), where PS was replaced by benzoic acid, did not show a similar bump. Instead, the bump in (b) was caused by the use of PS starting materials, and this broad peak was attributed to the degradation products of PS (most likely aromatic oligomers, as discussed in Figure S9).

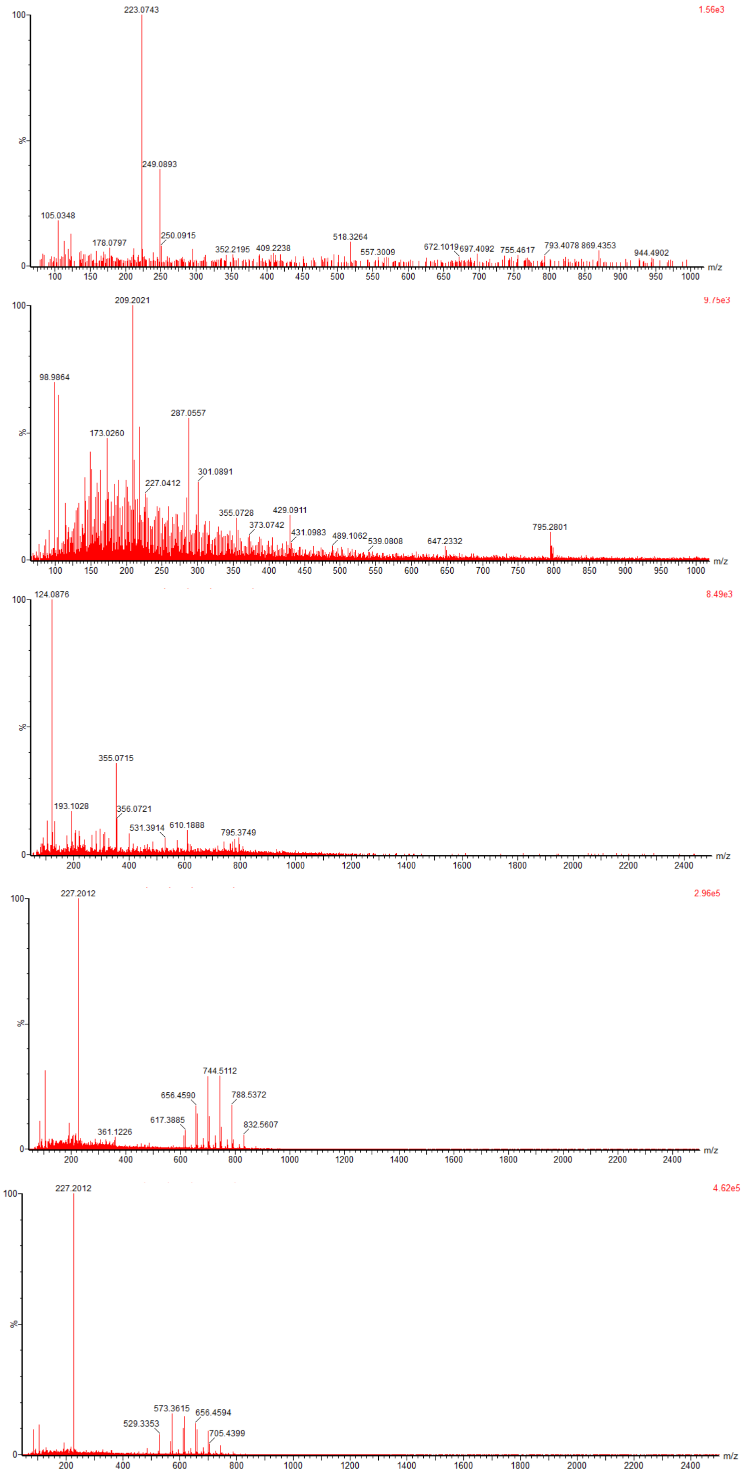

Figure S9. Mass spectrometry results for the reaction mixture of PS deconstruction after 24 h. Many peaks with molecular weights between 200 and 800 (e.g. 209, 223, 227, 249, 287, 355, 529, 573, 617, 656, 744, 788, 795, 832) were found, which were attributed to aromatic oligomers formed by PS deconstruction. Measurements were taken on a Xevo G2-S mass spectrometer with electrospray ionization method. Different spectra represent different peaks in the liquid chromatography prior to the mass spectroscopy measurement.

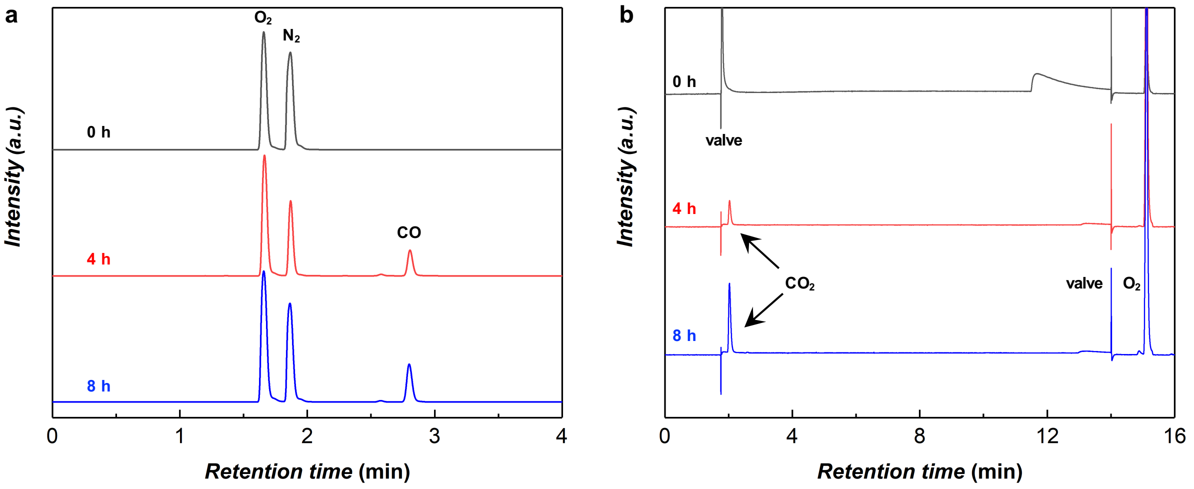

Figure S10. Gas chromatography detection for CO (**a**) and CO_2 (**b**) formed in PS deconstruction at 0 h (black), 4 h (red) and 8 h (blue). O_2, N_2, CO and CO_2 were labeled. CO was detected by barrier ionization discharge (BID) detector with He as the carrier gas. CO_2 was detected by thermal conductivity detector (TCD) with N_2 as the carrier gas. Reaction conditions: 0.1 M PS, 0.2 equiv. fluorenone, 1 equiv. H_2SO_4, 2 mL EtOAc, O_2 balloon, blue LED irradiation, 50 ± 3 °C.

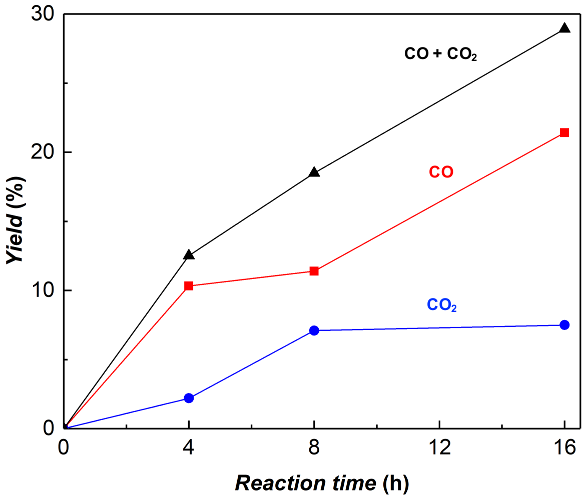

Figure S11. Production of CO_2 (blue) and CO (red) during PS deconstruction. The total amount of CO_2 and CO are also shown (black). Reaction conditions: 0.1 M PS, 0.2 equiv. fluorenone, 1 equiv. H_2SO_4, 2 mL EtOAc, O_2 balloon, blue LED irradiation, 50 ± 3 °C.

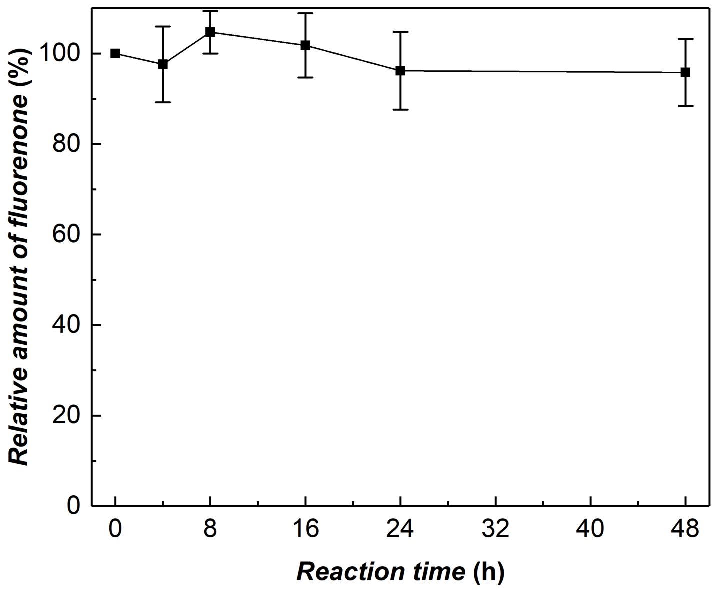

Figure S12. Changes of the relative amount of fluorenone photocatalyst over the reaction time. Reaction conditions: 0.1 M PS, 0.2 equiv. fluorenone, 1 equiv. H_2SO_4, 2 mL EtOAc, O_2 balloon, blue LED, 50 ± 3 °C (due to the heating from LED). Concentration of fluorenone was measured by HPLC and the concentration at 0 h is set as 100%. Error bars correspond to the standard deviation of triplicate experiments.

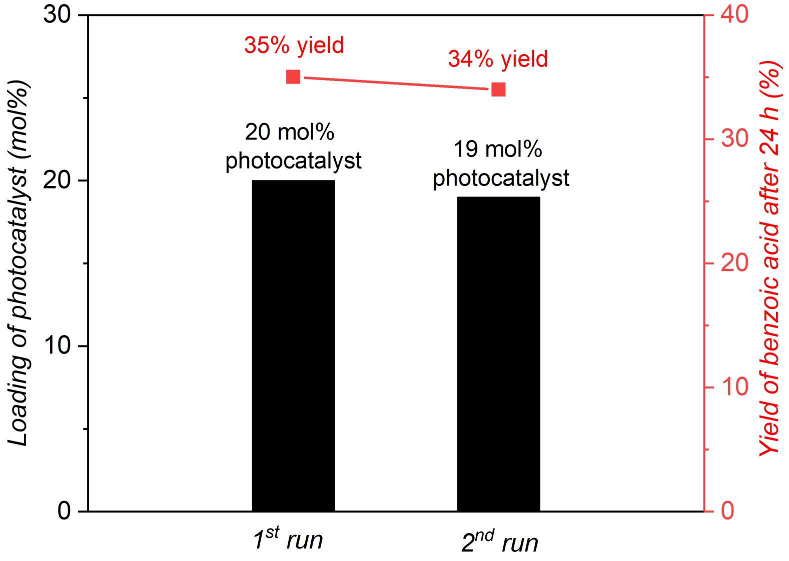

Figure S13. Reusability of the fluorenone photocatalyst. The 1st run of reaction used 20 mol% photocatalyst to drive deconstruction of 0.2 mmol PS and gave 35% yield of benzoic acid after 24 h. The photocatalyst was then isolated with a yield of 95% (i.e. 19 mol% photocatalyst). The 2nd run of reaction started with this recycled photocatalyst and gave 34% yield of benzoic acid after 24 h.

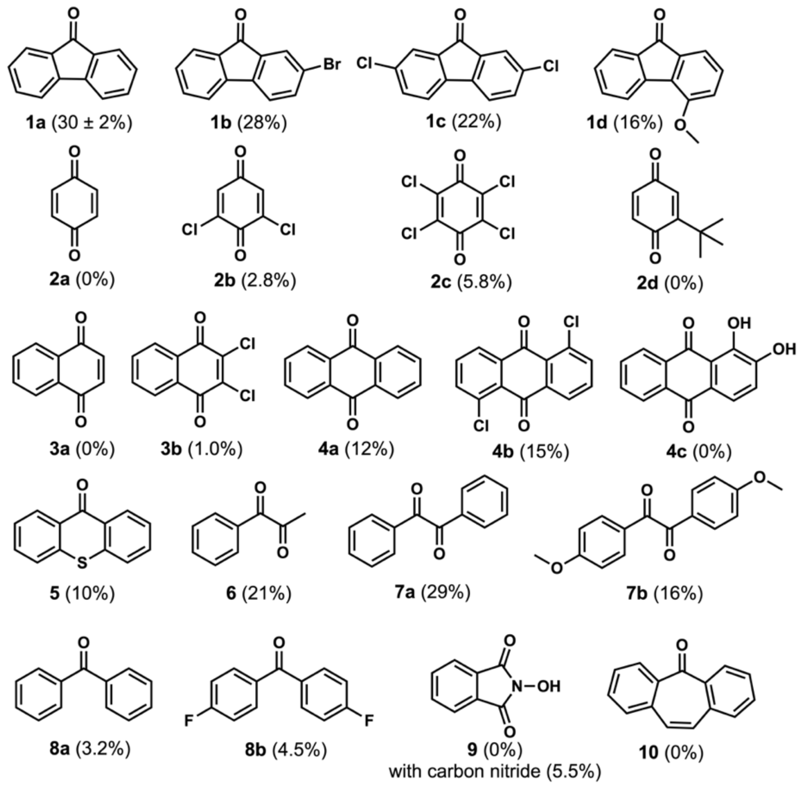

Figure S14. Different photocatalysts for PS deconstruction and the yields of benzoic acid at 16 h. Reaction conditions: 0.2 mmol PS, 1 equiv. H_2SO_4, 2 mL EtOAc, O_2 balloon, 16 h under blue LED, 50 ± 3 °C. Photocatalyst loading is 20 mol%, except **4a**, **4b** and **4c** (5 mol% due to the solubility limit). Concentration of benzoic acid was measured by HPLC analysis of the reaction mixture. Product yield is given in brackets and is calculated by the product concentration divided by the initial concentration of substrate (0.1 M).

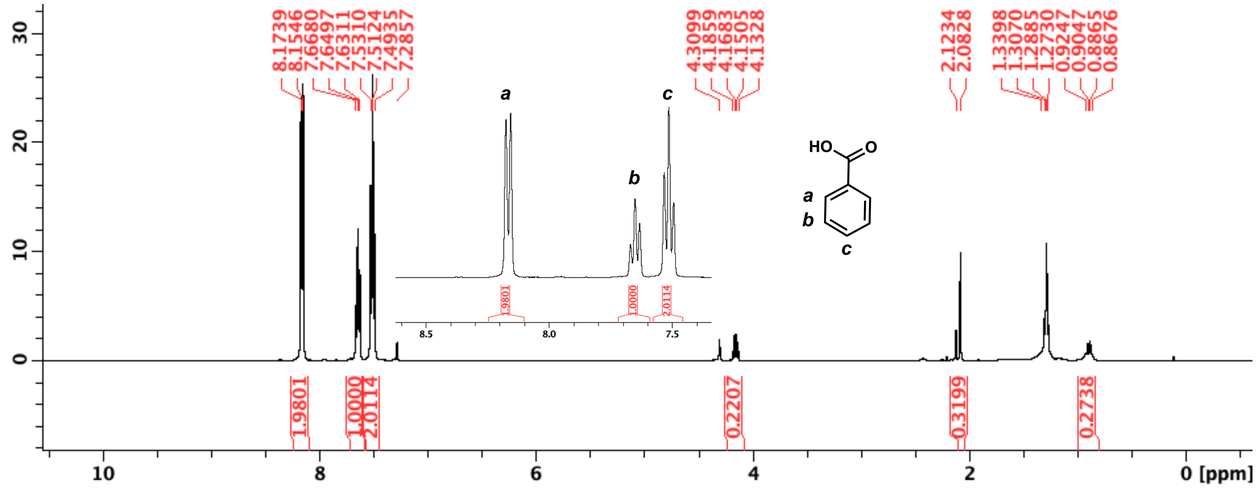

Figure S15. ^{1}H NMR spectra for isolated benzoic acid from gram-scale PS deconstruction reaction. Inset shows the aromatic region for benzoic acid. $CDCl_3$ was used as the deuterated reagent for the ^{1}H NMR measurement. The peak at 7.3 ppm is attributed to $CDCl_3$. The peaks at 4.1 ppm, 2.1 ppm and 0.9 ppm are attributed to remaining ethyl acetate solvent. The peak at 1.3 ppm is grease peak.

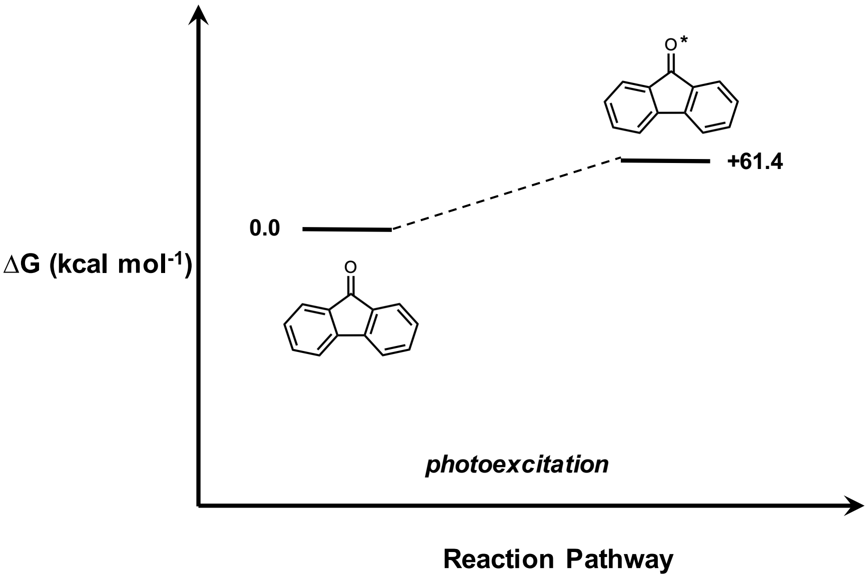

Figure S16. Energy diagram for the photoexcitation step of the fluorenone photocatalyst (level of theory: B3LYP/6-31G*//B3LYP/cc-pVTZ; ethyl acetate solvent PCM-SMD and Grimme D3 dispersions). The energy of the ground state of fluorenone is set to zero energy. The energy change corresponds to the π→π* adiabatic excitation, which is driven by the blue LED irradiation.

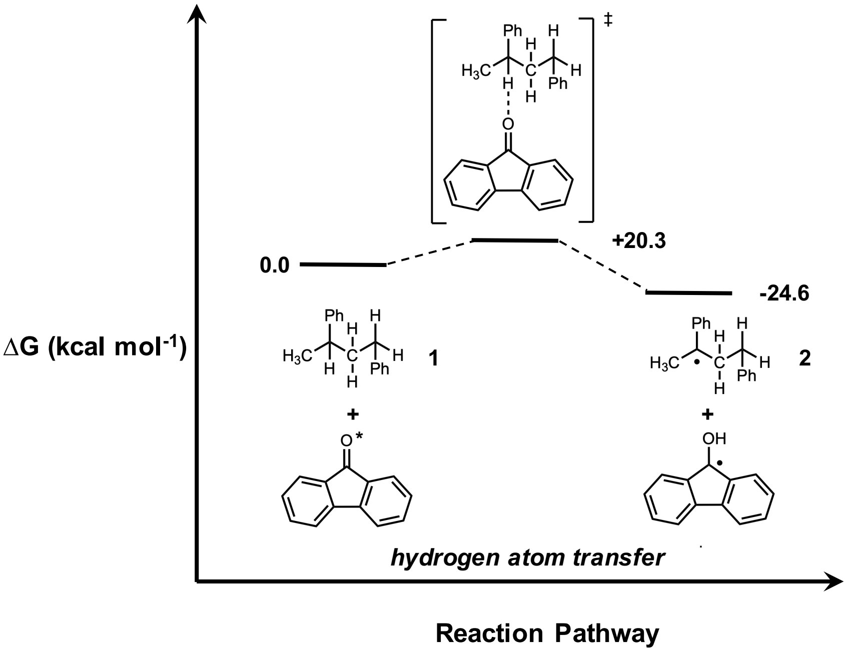

Figure S17. Energy diagram for the hydrogen atom transfer step, where the benzylic hydrogen in the styrene dimer (compound 1) is abstracted by the photoexcited-fluorenone to form a styrene dimer radical (compound 2). Level of theory: B3LYP/6-31G*//B3LYP/cc-pVTZ; ethyl acetate solvent PCM-SMD and Grimme D3 dispersions. The total energy of the styrene dimer and the excited state of fluorenone is set to zero energy. The transition state was calculated based on triplet state of the excited fluorenone. The TS calculated as a singlet state would give a higher kinetic barrier (+30.6 kcal mol^{-1}).

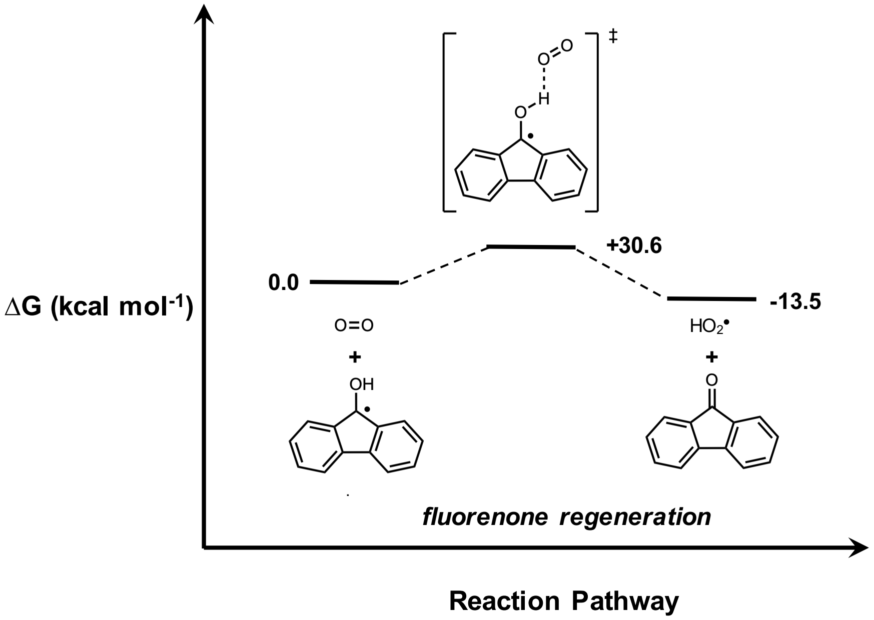

Figure S18. Energy diagram for the regeneration of fluorenone photocatalyst (level of theory: B3LYP/6-31G*//B3LYP/cc-pVTZ; ethyl acetate solvent PCM-SMD and Grimme D3 dispersions). The total energy of the O_2 and the fluorenone ketyl radical is set to zero energy.

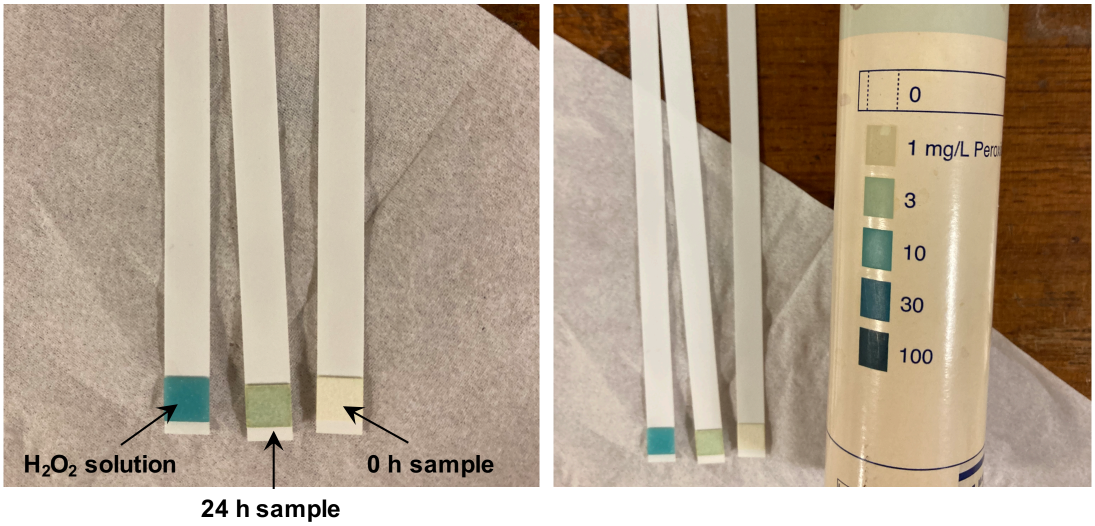

Figure S19. Detection of H_2O_2 by test strips. The strip immersed in H_2O_2 solution (left) showed a blue color. The strip immersed in the sample after 24 h reaction (middle) showed a green color, indicating formation of H_2O_2. The strip immersed in the sample before reaction (right) did not show any color.

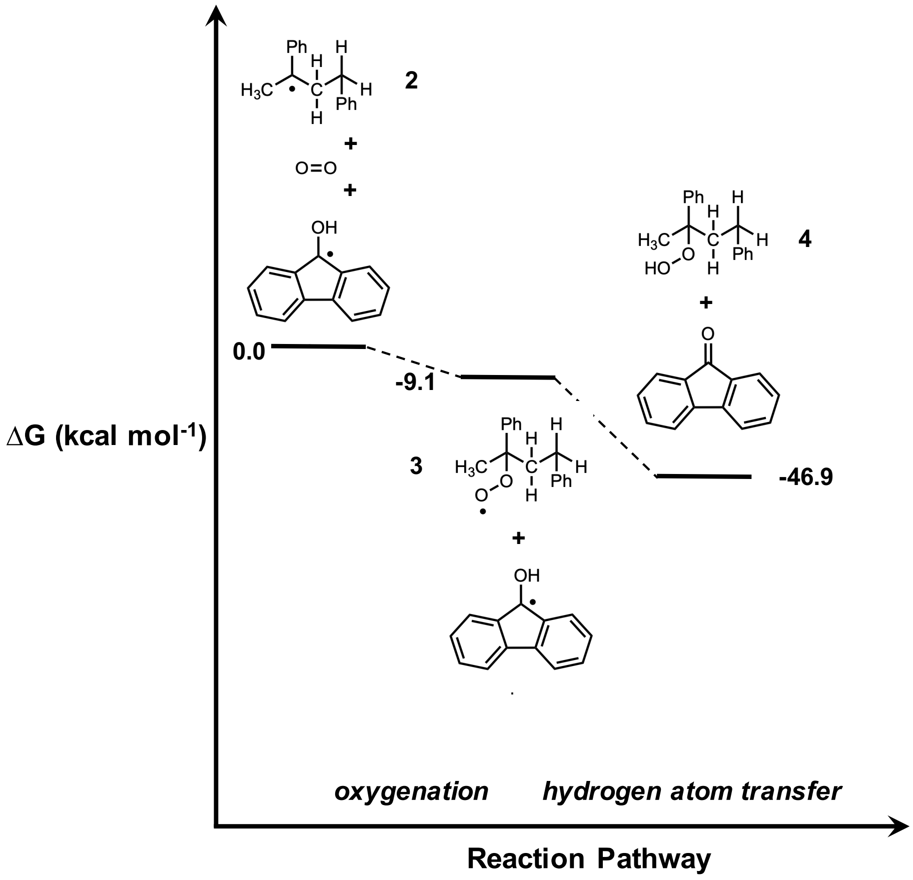

Figure S20. Energy diagram for the reaction pathway from styrene dimer radical (compound 2) to styrene dimer peroxide (compound 4). Level of theory: B3LYP/6-31G*//B3LYP/cc-pVTZ; ethyl acetate solvent PCM-SMD and Grimme D3 dispersions). The total energy of the styrene dimer radical, O_2 and the fluorenone ketyl radical is set to zero energy.

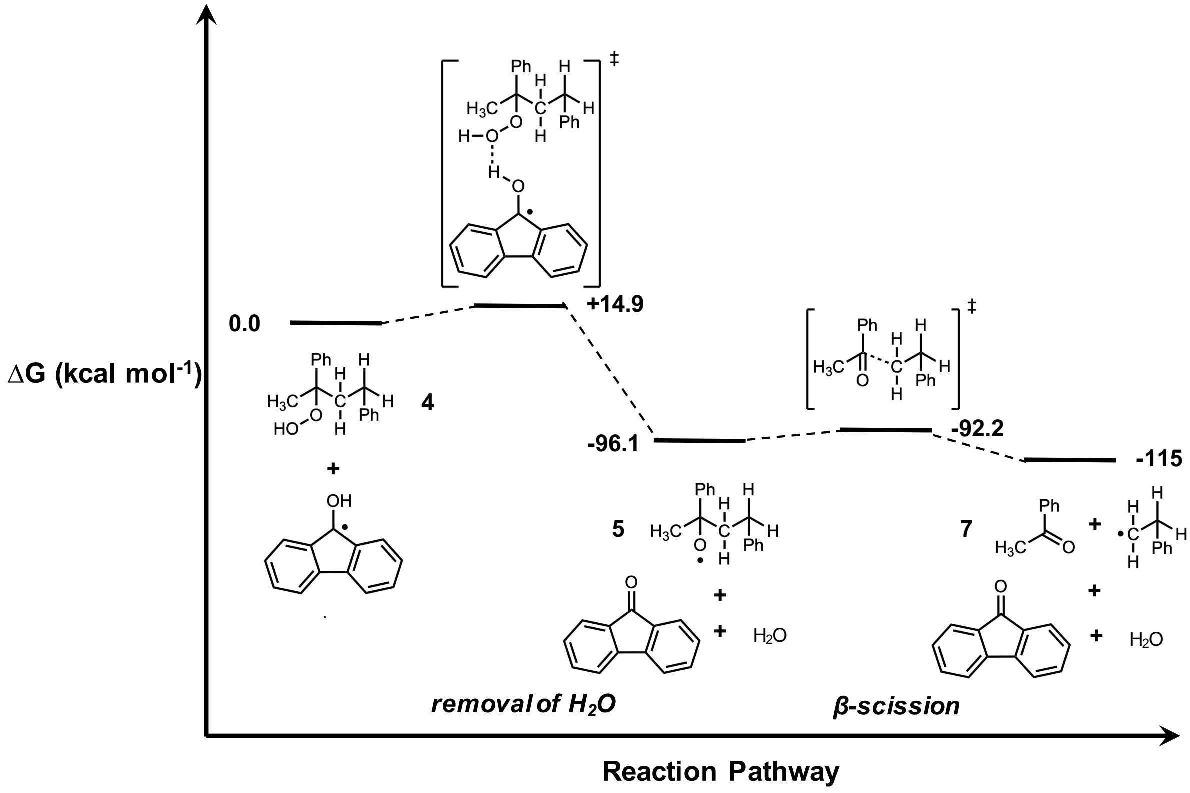

Figure S21. Energy diagram for the reaction pathway from styrene dimer peroxide (compound 4) to the C-C bond cleavage products (compounds 6 and 7). Level of theory: B3LYP/6-31G*//B3LYP/cc-pVTZ; ethyl acetate solvent PCM-SMD and Grimme D3 dispersions). The total energy of the styrene dimer peroxide and the fluorenone ketyl radical is set to zero energy.

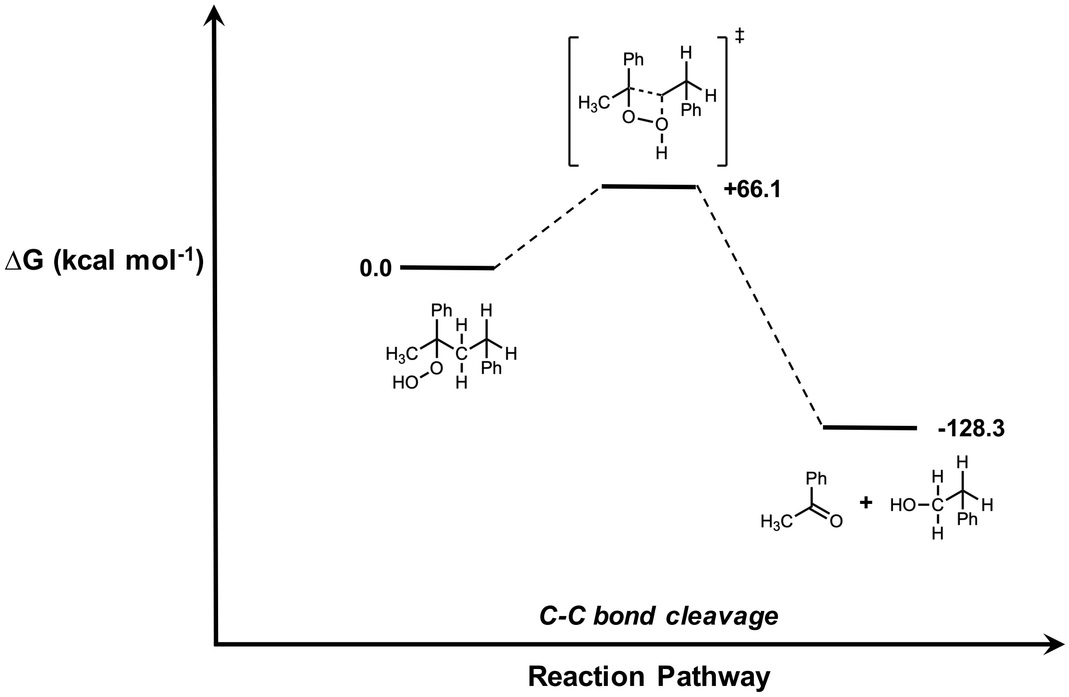

Figure S22. Energy diagram for the C-C bond cleavage reaction pathway without acid (level of theory: B3LYP/6-31G*//B3LYP/cc-pVTZ; ethyl acetate solvent PCM-SMD and Grimme D3 dispersions). The energy of the styrene dimer peroxide is set to zero energy.

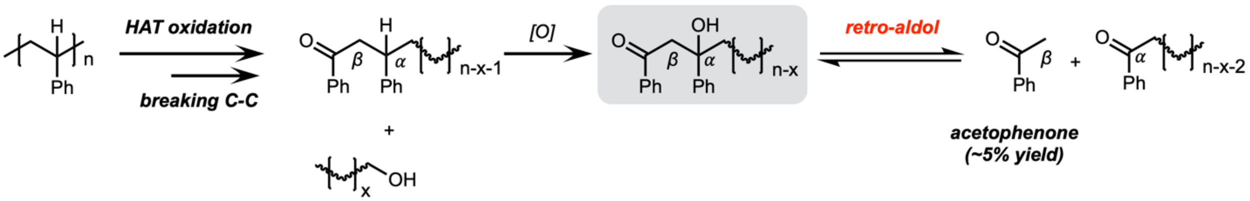

Figure S23. Alternative pathway for C-C bond cleavage. The steps of HAT oxidation and the cleavage of the first C-C in PS are similar to Figure 4a in main text, which generate a PS intermediate with a terminal aromatic C=O group. The H on alpha position in the next styrene monomer can be oxidized to form hydroxyl group. The highlighted compound can undergo retro-aldol reaction to break the C_α-C_β bond to generate acetophenone and a smaller PS intermediate. The acetophenone is detected with ~5% yield. However, this retro-aldol reaction is slow at room temperatures, which is consistent with the low yield of acetophenone.

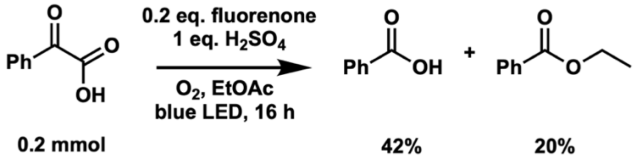

Figure S24. Conversion of phenylglyoxylic acid to benzoic acid and ethyl benzoate. Reaction conditions: 0.1 M phenylglyoxylic acid, 0.2 equiv. fluorenone, 1 equiv. H_2SO_4, 2 mL EtOAc, O_2 balloon, blue LED irradiation, 50 ± 3 °C. Concentrations of products were measured by HPLC and ^{1}H NMR analysis of the reaction mixture.

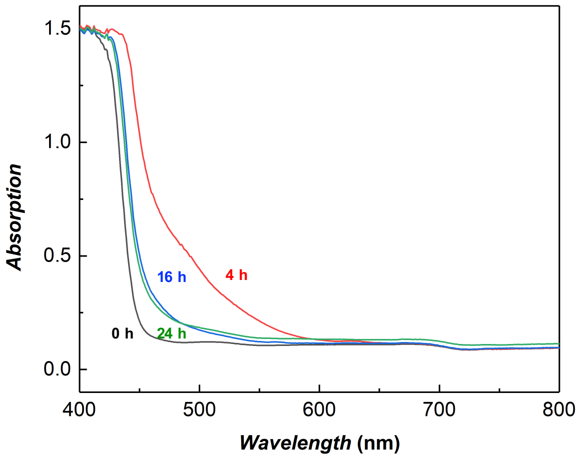

Figure S25. UV-Vis absorption for the reaction mixtures at 0 h (black), 4 h (red), 16 h (blue), and 24 h (green). Reaction conditions: 0.1 M PS, 0.2 equiv. fluorenone, 1 equiv. H_2SO_4, 2 mL EtOAc, O_2 balloon, blue LED irradiation, 50 ± 3 °C.

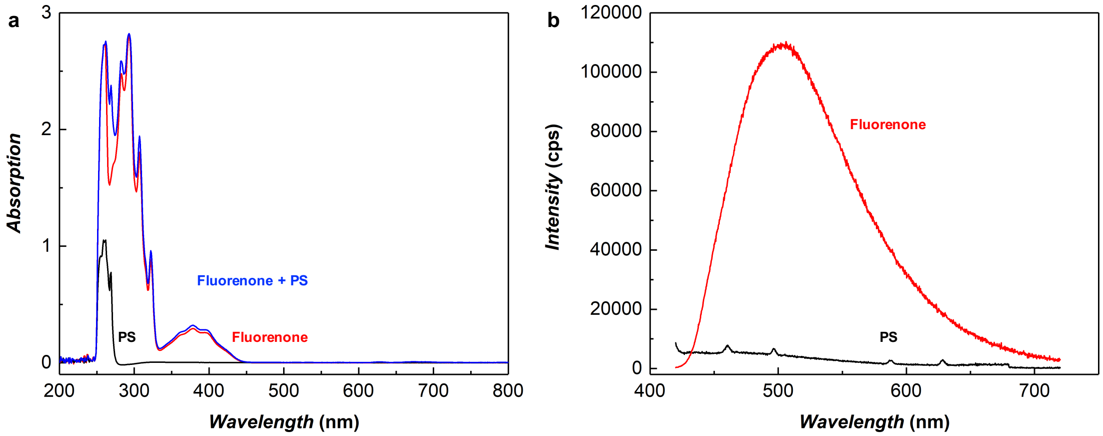

Figure S26. (a) UV-Vis absorption spectra for 5 mM PS (black), 1 mM fluorenone (red), and 1 mM fluorenone + 5 mM PS (blue). Baseline was collected with EtOAc solvent. (b) Fluorescence emission spectra for 100 mM PS (black) and 10 mM fluorenone (red) collected under N_2 atmosphere. Excitation wavelength was 405 nm. PS did not show any fluorescence around measuring region.

Table S1. Optimization of reaction conditions for PS deconstruction.[a]

entry	PS	fluorenone	solvent	acid	oxygen source	light source	benzoic acid yield [b]
1	0.05 M	0.2 equiv.	EtOAc	1 equiv. H_2SO_4	O_2 balloon	blue LED	23%
2	0.1 M	0.2 equiv.	EtOAc	1 equiv. H_2SO_4	O_2 balloon	blue LED	30±2% (38±3%) [c]
3	0.25 M	0.2 equiv.	EtOAc	1 equiv. H_2SO_4	O_2 balloon	blue LED	21%
4	0.5 M	0.2 equiv.	EtOAc	1 equiv. H_2SO_4	O_2 balloon	blue LED	8.7%
5	0.1 M	0.05 equiv.	EtOAc	1 equiv. H_2SO_4	O_2 balloon	blue LED	21%
6	0.1 M	0.1 equiv.	EtOAc	1 equiv. H_2SO_4	O_2 balloon	blue LED	24%
7	0.1 M	0.4 equiv.	EtOAc	1 equiv. H_2SO_4	O_2 balloon	blue LED	34%
8	0.1 M	0.2 equiv.	BuOAc	1 equiv. H_2SO_4	O_2 balloon	blue LED	8.4%
9	0.1 M	0.2 equiv.	CH_2Cl_2	1 equiv. H_2SO_4	O_2 balloon	blue LED	4.4%
10	0.1 M	0.2 equiv.	$CHCl_3$	1 equiv. H_2SO_4	O_2 balloon	blue LED	2.2%
11	0.1 M	0.2 equiv.	THF	1 equiv. H_2SO_4	O_2 balloon	blue LED	0%
12	0.1 M	0.2 equiv.	Acetonitrile	1 equiv. H_2SO_4	O_2 balloon	blue LED	0%
13	0.1 M	0.2 equiv.	Benzene	1 equiv. H_2SO_4	O_2 balloon	blue LED	0%
14	0.1 M	0.2 equiv.	1,4-Dioxane	1 equiv. H_2SO_4	O_2 balloon	blue LED	0%
15	0.1 M	0.2 equiv.	Butanone	1 equiv. H_2SO_4	O_2 balloon	blue LED	0%
16	0.1 M	0.2 equiv.	Cyclohexanone	1 equiv. H_2SO_4	O_2 balloon	blue LED	0%
17	0.1 M	0.2 equiv.	EtOAc +2% H_2O	1 equiv. H_2SO_4	O_2 balloon	blue LED	0%
18	0.1 M	0.2 equiv.	EtOAc	2 equiv. H_2SO_4	O_2 balloon	blue LED	35%
19	0.1 M	0.2 equiv.	EtOAc	0.5 equiv. H_2SO_4	O_2 balloon	blue LED	27%
20	0.1 M	0.2 equiv.	EtOAc	0.25 equiv. H_2SO_4	O_2 balloon	blue LED	22%
21	0.1 M	0.2 equiv.	EtOAc	2/3 equiv. H_3PO_4	O_2 balloon	blue LED	trace
22	0.1 M	0.2 equiv.	EtOAc	2 equiv. HNO_3	O_2 balloon	blue LED	8.5%
23	0.1 M	0.2 equiv.	EtOAc	2 equiv. $HClO_4$	O_2 balloon	blue LED	27%
24	0.1 M	0.2 equiv.	EtOAc	2 equiv. CF_3SO_3H	O_2 balloon	blue LED	16%
25	0.1 M	0.2 equiv.	EtOAc	2 equiv. HI	O_2 balloon	blue LED	0%
26	0.1 M	0.2 equiv.	EtOAc	1 equiv. H_2SO_4	air balloon	blue LED	8.8%
27	0.1 M	0.2 equiv.	EtOAc	1 equiv. H_2SO_4	$H_2O_{2\,(aq)}$	blue LED	0%
28	0.1 M	0.2 equiv.	EtOAc	1 equiv. H_2SO_4	$^{t}BuOOH_{(aq)}$	blue LED	0%
29	0.1 M	0.2 equiv.	EtOAc	1 equiv. H_2SO_4	O_2 balloon	AM 1.5G solar simulator	4.2% (22%) [d]

30	0.1 M	0.2 equiv.	EtOAc	1 equiv. H_2SO_4	O_2 balloon	UV lamp [e]	9.6%
31	0.1 M	0.2 equiv.	EtOAc	1 equiv. H_2SO_4	O_2 balloon	blue LED with a water bath at 25℃	17% (33%) [f]

[a] 2 mL solvent.
[b] Concentration of benzoic acid was quantified by HPLC analysis of the reaction mixture at 16 h. Product yield was calculated by the product concentration divided by the initial concentration of PS.
[c] 30±2% yield at 16 h, 38±3% yield at 48 h.
[d] 4.2% yield at 16 h, 22% yield at 48 h.
[e] UV lamp (1.2 W): wavelength = 405 nm.
[f] 17% yield at 16 h, 33% yield at 48 h.

Calculated cartesian coordinates for intermediates.

Fluorenone - Ground state (singlet, G = -575.535610)

C	0.00000000	3.02801700	-1.39537300
C	0.00000000	3.46686200	-0.06702500
C	0.00000000	2.53938100	0.98474600
C	0.00000000	1.18881800	0.66775400
C	0.00000000	0.74109400	-0.66958800
C	0.00000000	1.65965900	-1.71221700
H	0.00000000	3.75932300	-2.19928200
H	0.00000000	4.53145100	0.14888600
H	0.00000000	2.86211800	2.02224000
H	0.00000000	1.33363800	-2.74853900
C	0.00000000	0.00000000	1.57738300
C	0.00000000	-1.18881800	0.66775400
C	0.00000000	-2.53938100	0.98474600
C	0.00000000	-3.46686200	-0.06702500
C	0.00000000	-3.02801700	-1.39537300
C	0.00000000	-1.65965900	-1.71221700
C	0.00000000	-0.74109400	-0.66958800
H	0.00000000	-2.86211800	2.02224000
H	0.00000000	-4.53145100	0.14888600
H	0.00000000	-3.75932300	-2.19928200
H	0.00000000	-1.33363800	-2.74853900
O	0.00000000	0.00000000	2.79869200

Fluorenone – Excited State (singlet, G = -575.465416)

C	-0.05194400	-0.00033300	3.43062300
C	0.99777800	-0.00022500	2.52064000
C	0.70097700	0.00002000	1.15714500
C	-0.69710700	0.00015000	0.70158500
C	-1.75309500	0.00021000	1.66131100
H	-2.21129500	-0.00009500	3.74904400
H	0.15562500	-0.00061500	4.49698500
H	2.03217700	-0.00038500	2.85260000
H	-2.78883400	0.00039600	1.33382800
C	1.60416400	0.00020900	0.00000000
C	0.70097700	0.00002000	-1.15714500
C	0.99777800	-0.00022500	-2.52064000
C	-0.05194400	-0.00033300	-3.43062300
C	-1.42901700	-0.00005900	-2.99572200
C	-1.75309500	0.00021000	-1.66131100
C	-0.69710700	0.00015000	-0.70158500
H	2.03217700	-0.00038500	-2.85260000
H	0.15562500	-0.00061500	-4.49698500
H	-2.21129500	-0.00009500	-3.74904400
H	-2.78883400	0.00039600	-1.33382800
O	2.84856900	0.00037400	0.00000000

Compound 1 (singlet, G = -1196.088)

C	-3.414606000	-1.492614000	-2.218434000
H	-4.378495000	-2.013629000	-2.281608000
H	-2.623340000	-2.207084000	-2.478388000
C	-3.177380000	-0.913758000	-0.809722000
C	-3.103679000	-2.017197000	0.257334000
H	-4.069501000	-2.534635000	0.331758000
H	-2.377254000	-2.769885000	-0.076703000
C	-2.696829000	-1.517713000	1.663662000
H	-2.818635000	-2.351556000	2.366432000
H	-3.393710000	-0.736186000	1.984159000
H	-2.184215000	-0.443116000	-0.835961000
H	-3.412241000	-0.697180000	-2.972586000
C	-1.276972000	-0.996130000	1.775485000
C	-0.216264000	-1.887693000	2.000445000
C	-0.982207000	0.373261000	1.685716000
C	1.092628000	-1.429242000	2.155983000
H	-0.425897000	-2.953172000	2.070909000
C	0.328508000	0.837946000	1.837028000
H	-1.789029000	1.080441000	1.506307000
C	1.369262000	-0.061198000	2.080828000
H	1.896789000	-2.138274000	2.333120000
H	0.535843000	1.902873000	1.767785000
H	2.386298000	0.299444000	2.201296000
C	-4.162330000	0.211802000	-0.497001000
C	-3.871488000	1.479055000	-1.025519000
C	-5.307875000	0.083652000	0.305091000
C	-4.669196000	2.587923000	-0.749967000
H	-2.985457000	1.597910000	-1.646250000
C	-6.108440000	1.196268000	0.593529000
H	-5.569063000	-0.880335000	0.733091000
C	-5.790497000	2.452565000	0.073086000
H	-4.407252000	3.558483000	-1.164120000
H	-6.979364000	1.078077000	1.233781000
H	-6.407298000	3.316441000	0.307189000
C	3.100432000	-3.186746000	-0.667560000
C	1.935220000	-2.987232000	-1.415592000
C	1.517109000	-1.689168000	-1.743307000
C	2.286658000	-0.622828000	-1.302540000
C	3.456748000	-0.818152000	-0.540142000
C	3.875687000	-2.103530000	-0.221436000
H	3.408855000	-4.198877000	-0.419730000
H	1.350140000	-3.843044000	-1.739915000
H	0.609389000	-1.515633000	-2.315141000
H	4.771660000	-2.272464000	0.369392000
C	2.047979000	0.846371000	-1.456950000
C	3.156915000	1.504905000	-0.696335000
C	3.388038000	2.853323000	-0.467435000
C	4.497545000	3.212831000	0.311626000
C	5.341874000	2.227916000	0.835233000
C	5.102984000	0.863096000	0.603048000
C	4.001225000	0.507820000	-0.165525000
H	2.719411000	3.604193000	-0.879444000
H	4.703183000	4.260523000	0.511619000
H	6.197459000	2.522618000	1.436980000
H	5.762779000	0.108162000	1.021548000
O	1.144084000	1.391589000	-2.071163000

Compound 2 (triplet, G = -1196.03499)

C	-2.665551000	-1.429446000	-2.251415000
H	-3.516281000	-1.965084000	-2.697854000
H	-1.983601000	-2.205752000	-1.875122000
C	-3.150465000	-0.547771000	-1.134110000
C	-4.008164000	-1.237014000	-0.096495000
H	-4.965159000	-0.710130000	0.029226000
H	-4.263175000	-2.236331000	-0.465601000
C	-3.367323000	-1.380530000	1.310889000
H	-4.066876000	-1.956071000	1.933239000
H	-3.280613000	-0.393804000	1.776232000
H	-0.601003000	-0.541866000	-0.534369000
H	-2.154601000	-0.898504000	-3.056581000
C	-2.005931000	-2.046379000	1.328584000
C	-1.791432000	-3.294113000	0.719615000
C	-0.923915000	-1.431616000	1.974562000
C	-0.534231000	-3.899100000	0.748323000
H	-2.615696000	-3.795216000	0.218212000
C	0.335635000	-2.037671000	2.011965000
H	-1.065774000	-0.464360000	2.449574000
C	0.536058000	-3.271956000	1.393904000
H	-0.389338000	-4.862289000	0.265132000
H	1.159603000	-1.535303000	2.511235000
H	1.516688000	-3.740130000	1.409597000
C	-2.848957000	0.848594000	-1.049360000
C	-1.916514000	1.487772000	-1.925335000
C	-3.461539000	1.686562000	-0.064717000
C	-1.621068000	2.839491000	-1.815051000
H	-1.398522000	0.905301000	-2.677692000
C	-3.159804000	3.038585000	0.033294000
H	-4.198000000	1.269514000	0.612534000
C	-2.235197000	3.632165000	-0.836334000
H	-0.895458000	3.281301000	-2.493424000
H	-3.652012000	3.639616000	0.794045000
H	-1.995876000	4.688258000	-0.751143000
C	5.378104000	-0.701270000	-1.097181000
C	4.522894000	-1.579453000	-1.786091000
C	3.136918000	-1.468227000	-1.669593000
C	2.613970000	-0.457362000	-0.850397000
C	3.482639000	0.434322000	-0.144051000
C	4.862249000	0.307898000	-0.271741000
H	6.454300000	-0.808167000	-1.203510000
H	4.948693000	-2.355719000	-2.416481000
H	2.474880000	-2.148668000	-2.197802000
H	5.532381000	0.977653000	0.261550000
C	1.264275000	-0.107482000	-0.522113000
C	1.274332000	1.003867000	0.386203000
C	0.236995000	1.723312000	0.999927000
C	0.568366000	2.774992000	1.854214000
C	1.910754000	3.113100000	2.098122000
C	2.953585000	2.403378000	1.485666000
C	2.643674000	1.350443000	0.631008000
H	-0.803200000	1.478894000	0.810926000
H	-0.225033000	3.343510000	2.332030000
H	2.143737000	3.937398000	2.766797000
H	3.988978000	2.674236000	1.676972000
O	0.208600000	-0.763602000	-1.031436000

Compound 2 (singlet, G = -1195.57611)

C	2.696344000	-1.423065000	2.257653000
H	3.504155000	-2.161057000	2.527397000
H	1.787408000	-2.036615000	1.945042000
C	3.178169000	-0.546888000	1.137496000
C	4.020543000	-1.231378000	0.081557000
H	4.976489000	-0.702524000	-0.053792000
H	4.282640000	-2.231430000	0.437152000
C	3.362965000	-1.361528000	-1.320318000
H	4.057529000	-1.928965000	-1.955442000
H	3.273982000	-0.373462000	-1.777395000
H	0.593128000	-0.548011000	0.502083000
H	2.418288000	-0.841138000	3.178871000
C	2.002622000	-2.029516000	-1.333170000
C	1.791052000	-3.269724000	-0.708098000
C	0.923597000	-1.429189000	-1.990424000
C	0.534779000	-3.881561000	-0.741831000
H	2.611941000	-3.761300000	-0.189734000
C	-0.330098000	-2.037116000	-2.032843000
H	1.068123000	-0.465068000	-2.477383000
C	-0.532553000	-3.263874000	-1.403594000
H	0.392170000	-4.841637000	-0.246683000
H	-1.155620000	-1.544321000	-2.539171000
H	-1.512448000	-3.738513000	-1.419341000
C	2.859456000	0.844320000	1.047824000
C	1.917432000	1.469281000	1.922297000
C	3.464265000	1.691365000	0.069941000
C	1.609783000	2.815895000	1.821768000
H	1.396564000	0.873687000	2.663632000
C	3.155275000	3.043203000	-0.023242000
H	4.210253000	1.282648000	-0.609758000
C	2.221157000	3.622583000	0.849671000
H	0.879546000	3.249412000	2.497700000
H	3.640426000	3.652397000	-0.777818000
H	1.975074000	4.678552000	0.768259000
C	-5.374933000	-0.713957000	1.103042000
C	-4.517281000	-1.592985000	1.787825000
C	-3.131669000	-1.479145000	1.664658000
C	-2.616513000	-0.464828000	0.852898000
C	-3.487680000	0.432682000	0.150758000
C	-4.866884000	0.303617000	0.285059000
H	-6.450787000	-0.822939000	1.215589000
H	-4.940886000	-2.371964000	2.416345000
H	-2.467747000	-2.160224000	2.189670000
H	-5.538891000	0.974014000	-0.245058000
C	-1.267884000	-0.116639000	0.513809000
C	-1.281089000	1.002548000	-0.385935000
C	-0.245831000	1.725633000	-0.998892000
C	-0.580173000	2.779531000	-1.849279000
C	-1.923467000	3.121200000	-2.085113000
C	-2.964236000	2.407833000	-1.473385000
C	-2.651352000	1.352644000	-0.622599000
H	0.795038000	1.477346000	-0.817207000
H	0.211594000	3.350860000	-2.326449000
H	-2.158777000	3.947277000	-2.750772000
H	-4.000341000	2.677630000	-1.657313000
O	-0.205454000	-0.782540000	1.004547000

TS 1-2 (triplet, G = -1195.9587)

C	-1.976037000	-1.339757000	-1.610916000
H	-2.300182000	-2.101699000	-2.339551000
H	-1.102434000	-1.760768000	-1.097676000
C	-3.071554000	-1.066908000	-0.621664000
C	-3.326152000	-2.183752000	0.362336000
H	-4.391687000	-2.448500000	0.404789000
H	-2.800584000	-3.082488000	0.018806000
C	-2.843095000	-1.875712000	1.808732000
H	-3.071371000	-2.750212000	2.431957000
H	-3.416735000	-1.034661000	2.211366000
H	-0.867437000	0.957123000	-0.353254000
H	-1.636217000	-0.466829000	-2.169466000
C	-1.365147000	-1.558896000	1.885628000
C	-0.405289000	-2.573734000	1.747954000
C	-0.918726000	-0.240357000	2.054175000
C	0.959789000	-2.281948000	1.785860000
H	-0.733906000	-3.602049000	1.610281000
C	0.447851000	0.056299000	2.100680000
H	-1.647806000	0.558769000	2.161427000
C	1.390593000	-0.964445000	1.969102000
H	1.688705000	-3.080306000	1.670866000
H	0.773389000	1.084591000	2.230898000
H	2.450892000	-0.733923000	1.999103000
C	-3.839222000	0.137544000	-0.602814000
C	-3.522416000	1.262944000	-1.425479000
C	-4.970252000	0.287798000	0.259336000
C	-4.267428000	2.433532000	-1.377681000
H	-2.673807000	1.216183000	-2.098065000
C	-5.709950000	1.462558000	0.295740000
H	-5.273590000	-0.535965000	0.896212000
C	-5.367908000	2.551339000	-0.517429000
H	-3.987014000	3.268224000	-2.015901000
H	-6.565168000	1.532497000	0.963903000
H	-5.946739000	3.470125000	-0.483037000
C	3.759175000	-2.614927000	-1.501873000
C	2.510670000	-2.594529000	-2.133205000
C	1.704853000	-1.448666000	-2.076747000
C	2.177223000	-0.346047000	-1.376461000
C	3.440858000	-0.360700000	-0.742660000
C	4.239011000	-1.495998000	-0.801069000
H	4.369645000	-3.512454000	-1.553514000
H	2.163957000	-3.473252000	-2.669530000
H	0.735248000	-1.419605000	-2.563328000
H	5.208380000	-1.525915000	-0.311371000
C	1.526654000	0.961271000	-1.108778000
C	2.484495000	1.728097000	-0.274193000
C	2.356635000	2.994910000	0.280771000
C	3.408813000	3.481964000	1.068777000
C	4.552198000	2.703550000	1.282623000
C	4.677921000	1.421803000	0.721467000
C	3.635220000	0.937140000	-0.058584000
H	1.459868000	3.583404000	0.108029000
H	3.337586000	4.468348000	1.518156000
H	5.358001000	3.096002000	1.897095000
H	5.568959000	0.826573000	0.901273000
O	0.395921000	1.327569000	-1.466405000

TS 1-2 (singlet, G = -1195.9423)

C	-3.366013000	-1.460385000	-2.232257000
H	-4.303084000	-2.013449000	-2.434331000
H	-2.533237000	-2.140815000	-2.422163000
C	-3.425850000	-1.013182000	-0.808040000
C	-3.090935000	-2.005827000	0.251959000
H	-4.017780000	-2.600170000	0.375486000
H	-2.344573000	-2.710341000	-0.129008000
C	-2.695596000	-1.493576000	1.661543000
H	-2.829575000	-2.338320000	2.346453000
H	-3.399082000	-0.717232000	1.976084000
H	-0.943000000	0.192294000	-1.317720000
H	-3.340842000	-0.621563000	-2.931068000
C	-1.276761000	-0.987001000	1.783308000
C	-0.223877000	-1.885558000	2.009744000
C	-0.980941000	0.380269000	1.688504000
C	1.086881000	-1.431194000	2.160282000
H	-0.438670000	-2.949898000	2.081944000
C	0.329152000	0.840606000	1.846893000
H	-1.785457000	1.087412000	1.503957000
C	1.365567000	-0.063306000	2.087195000
H	1.890060000	-2.142000000	2.333587000
H	0.540922000	1.904028000	1.772556000
H	2.384344000	0.293528000	2.203170000
C	-4.199552000	0.190273000	-0.492676000
C	-3.869234000	1.447990000	-1.037230000
C	-5.332623000	0.091714000	0.343499000
C	-4.646523000	2.566192000	-0.755729000
H	-2.960489000	1.528770000	-1.627104000
C	-6.119788000	1.212755000	0.603008000
H	-5.613800000	-0.871427000	0.758254000
C	-5.777297000	2.453403000	0.061501000
H	-4.361963000	3.532233000	-1.163197000
H	-6.999918000	1.114407000	1.232409000
H	-6.380375000	3.329939000	0.281532000
C	3.095796000	-3.190917000	-0.657841000
C	1.931705000	-2.991143000	-1.407197000
C	1.517761000	-1.693610000	-1.743975000
C	2.291171000	-0.626751000	-1.309621000
C	3.455880000	-0.821939000	-0.538485000
C	3.870994000	-2.107155000	-0.212841000
H	3.402983000	-4.202815000	-0.406884000
H	1.344687000	-3.847276000	-1.727820000
H	0.607451000	-1.514778000	-2.308077000
H	4.763853000	-2.275384000	0.383135000
C	2.049383000	0.840973000	-1.461028000
C	3.150350000	1.500230000	-0.687071000
C	3.384933000	2.848664000	-0.463055000
C	4.495499000	3.208964000	0.314134000
C	5.339649000	2.224571000	0.839276000
C	5.100201000	0.859844000	0.607868000
C	3.997614000	0.503728000	-0.159665000
H	2.717032000	3.599023000	-0.877358000
H	4.702236000	4.257056000	0.511553000
H	6.196098000	2.519734000	1.439683000
H	5.761332000	0.105120000	1.024956000
O	1.149445000	1.386265000	-2.079004000

Fluorenol-O_2 (doublet, G = -726.4986)

C	-1.487346000	2.466929000	-0.043236000
C	-2.321946000	1.336337000	-0.066169000
C	-1.784918000	0.048916000	-0.062309000
C	-0.389121000	-0.096325000	-0.036370000
C	0.462456000	1.056273000	-0.012341000
C	-0.092037000	2.331386000	-0.015572000
H	-1.929982000	3.459125000	-0.046654000
H	-3.399901000	1.468857000	-0.087358000
H	-2.441316000	-0.818456000	-0.078818000
H	0.545541000	3.211678000	0.002305000
C	0.454086000	-1.256458000	-0.029039000
C	1.825382000	-0.844602000	-0.000235000
C	3.019585000	-1.580167000	0.014625000
C	4.228695000	-0.884290000	0.040643000
C	4.253355000	0.521269000	0.052321000
C	3.063823000	1.263121000	0.037448000
C	1.848733000	0.586575000	0.011110000
H	3.001833000	-2.666366000	0.005755000
H	5.165161000	-1.435618000	0.052437000
H	5.207955000	1.040034000	0.073020000
H	3.093084000	2.349795000	0.046136000
O	0.086554000	-2.552308000	-0.047075000
H	-0.885674000	-2.617341000	-0.068781000
O	-5.774679000	-0.318853000	0.197464000
O	-5.167021000	-1.339525000	-0.055801000

Fluorenone-H_2O (doublet, G = -726.5202)

C	0.830592000	3.285510000	0.015073000
C	1.990719000	2.505712000	0.001198000
C	1.906860000	1.105562000	-0.008463000
C	0.643799000	0.528583000	-0.002465000
C	-0.531676000	1.312406000	0.007284000
C	-0.446009000	2.696826000	0.017038000
H	0.915066000	4.368825000	0.022930000
H	2.964090000	2.987473000	-0.003021000
H	2.802175000	0.489471000	-0.027104000
H	-1.339275000	3.314645000	0.025315000
C	0.251599000	-0.906469000	-0.007036000
C	-1.235323000	-0.924871000	-0.008310000
C	-2.103776000	-2.007376000	-0.015954000
C	-3.481693000	-1.746437000	-0.013858000
C	-3.951071000	-0.429092000	-0.003352000
C	-3.067110000	0.663802000	0.005203000
C	-1.703668000	0.405898000	0.002335000
H	-1.721431000	-3.024034000	-0.023799000
H	-4.189112000	-2.570453000	-0.020458000
H	-5.021863000	-0.244442000	-0.001750000
H	-3.446941000	1.681353000	0.013510000
O	0.982508000	-1.898743000	-0.006135000
H	2.673121000	-2.055745000	0.080141000
O	3.636290000	-2.338121000	0.139464000
O	4.349291000	-1.249063000	-0.133070000

TS Fluorenol-O_2 - Fluorenone-H_2O (doublet, G = -726.4498)

C	-1.470020000	2.479869000	-0.006743000
C	-2.315300000	1.365939000	-0.048017000
C	-1.779810000	0.070701000	-0.083634000
C	-0.398014000	-0.066334000	-0.072490000
C	0.460824000	1.055450000	-0.022152000
C	-0.071853000	2.337315000	0.006779000
H	-1.902208000	3.476534000	0.016514000
H	-3.391725000	1.508839000	-0.055954000
H	-2.418621000	-0.806920000	-0.120898000
H	0.570917000	3.212413000	0.043220000
C	0.424939000	-1.294648000	-0.079642000
C	1.836597000	-0.846243000	-0.018483000
C	3.008246000	-1.592669000	0.000283000
C	4.229881000	-0.906585000	0.041065000
C	4.256520000	0.492375000	0.058454000
C	3.070389000	1.245086000	0.037973000
C	1.858550000	0.567802000	-0.000016000
H	2.971504000	-2.678353000	-0.015963000
H	5.162589000	-1.463069000	0.058557000
H	5.212617000	1.008008000	0.088114000
H	3.106173000	2.330849000	0.052980000
O	0.015725000	-2.469723000	-0.103482000
H	-1.001546000	-2.881629000	1.113562000
O	-5.746093000	-0.325893000	0.173973000
O	-5.141556000	-1.348762000	-0.078042000

Compound 3 (doublet, G = -770.353154)

C	-0.13544600	1.17973000	-1.52587400
H	0.43166600	1.02280700	-2.44882100
H	-0.62625200	2.15471600	-1.57282500
C	0.79092700	1.14797600	-0.31563100
C	0.08642300	1.46730700	1.02114600
H	0.85956400	1.77869400	1.73268300
H	-0.56800200	2.33005400	0.86380500
C	-0.70293000	0.30916400	1.66736000
H	-1.03215800	0.66852300	2.65122300
H	-0.02505000	-0.53042300	1.85419500
H	-0.90726200	0.41271200	-1.45060900
C	-1.90914700	-0.17285600	0.88933600
C	-3.00003100	0.68067100	0.66409100
C	-1.95769100	-1.46981800	0.36108100
C	-4.10202400	0.25509900	-0.07860300
H	-2.98054200	1.68961900	1.07049800
C	-3.05923300	-1.90097300	-0.38404000
H	-1.11931200	-2.14234200	0.52453700
C	-4.13428400	-1.03856200	-0.60919800
H	-4.93655600	0.93218000	-0.24330700
H	-3.07526200	-2.91014500	-0.78819800
H	-4.99121400	-1.37067800	-1.18948300
C	1.67422100	-0.08544400	-0.21426300
C	1.38930300	-1.25907000	-0.92372300
C	2.78045500	-0.07417500	0.65103000
C	2.18845200	-2.39549500	-0.77057800
H	0.53658300	-1.30164300	-1.59179200
C	3.58444000	-1.20437700	0.79624500
H	3.01923000	0.82738900	1.20701300
C	3.28895400	-2.37208100	0.08713200
H	1.94792300	-3.29787500	-1.32651900
H	4.44017000	-1.17362800	1.46556600
H	3.91182700	-3.25504300	0.20325300
O	1.80118400	2.26173300	-0.53645100
O	1.25135600	3.45858000	-0.57033400

Compound 4 (doublet, G = -1346.9877)

C	-4.432105000	-2.655782000	0.127215000
C	-3.646016000	-2.805707000	1.283182000
C	-2.926131000	-1.731190000	1.804936000
C	-3.000999000	-0.490536000	1.154118000
C	-3.806542000	-0.336801000	-0.020949000
C	-4.515971000	-1.420108000	-0.529054000
H	-4.981407000	-3.508656000	-0.262210000
H	-3.596946000	-3.773557000	1.775119000
H	-2.314373000	-1.857328000	2.693655000
H	-5.126076000	-1.311514000	-1.422383000
C	-2.378951000	0.776380000	1.426173000
C	-2.805090000	1.724160000	0.437592000
C	-2.472795000	3.072948000	0.244077000
C	-3.021994000	3.743619000	-0.849312000
C	-3.885698000	3.084109000	-1.741678000
C	-4.217536000	1.734128000	-1.558356000
C	-3.682096000	1.050775000	-0.471092000
H	-1.799626000	3.579117000	0.930323000
H	-2.777305000	4.789268000	-1.016659000
H	-4.299249000	3.627274000	-2.587135000
H	-4.879927000	1.230431000	-2.258072000
O	-1.537356000	1.097452000	2.419966000
H	-1.065116000	0.296453000	2.746453000
C	2.599156000	-1.578130000	1.256540000
H	2.423819000	-2.655724000	1.172743000
H	2.741902000	-1.327994000	2.311298000
C	1.402767000	-0.800941000	0.710012000
C	1.526846000	0.731162000	0.899704000
H	0.517804000	1.149096000	0.831219000
H	1.878049000	0.925772000	1.918339000
C	2.418425000	1.484329000	-0.110375000
H	2.296767000	2.554712000	0.102243000
H	2.034133000	1.323626000	-1.122998000
H	3.520099000	-1.327735000	0.727704000
C	3.891584000	1.137178000	-0.067318000
C	4.657660000	1.412389000	1.075867000
C	4.518980000	0.514469000	-1.154756000
C	6.007953000	1.065280000	1.135421000
H	4.188231000	1.898929000	1.928165000
C	5.871251000	0.163999000	-1.100096000
H	3.938112000	0.290291000	-2.046095000
C	6.620309000	0.436062000	0.046755000
H	6.583669000	1.286336000	2.030925000
H	6.337546000	-0.321761000	-1.953757000
H	7.671595000	0.163675000	0.092439000
C	1.026183000	-1.150914000	-0.730662000
C	1.871646000	-1.893637000	-1.563924000
C	-0.190052000	-0.680644000	-1.253452000
C	1.512669000	-2.156808000	-2.889331000
H	2.821251000	-2.263098000	-1.193760000
C	-0.553368000	-0.951543000	-2.571820000
H	-0.858586000	-0.106338000	-0.623506000
C	0.299164000	-1.690315000	-3.397178000
H	2.185577000	-2.729739000	-3.522065000
H	-1.502292000	-0.582632000	-2.952196000
H	0.020093000	-1.899081000	-4.426656000
O	0.200382000	-1.263224000	1.415676000
O	0.290121000	-0.885283000	2.830335000
H	0.281575000	-1.768978000	3.245616000

Compound 5 (doublet, G = -1347.1409)

C	-4.946160000	-1.530468000	-2.044914000
C	-4.251268000	-2.693495000	-1.698331000
C	-3.355380000	-2.691316000	-0.619241000
C	-3.185850000	-1.507757000	0.085677000
C	-3.873206000	-0.327469000	-0.269419000
C	-4.760447000	-0.330957000	-1.337074000
H	-5.637610000	-1.550675000	-2.883064000
H	-4.404132000	-3.603405000	-2.271475000
H	-2.777595000	-3.573844000	-0.363578000
H	-5.297504000	0.568481000	-1.624610000
C	-2.312368000	-1.215529000	1.259678000
C	-2.518482000	0.230086000	1.563222000
C	-1.902935000	1.022539000	2.521354000
C	-2.217466000	2.388702000	2.550582000
C	-3.128563000	2.922285000	1.632893000
C	-3.750639000	2.115110000	0.664953000
C	-3.438873000	0.761953000	0.636305000
H	-1.190688000	0.591061000	3.218977000
H	-1.748692000	3.037579000	3.284496000
H	-3.358860000	3.983741000	1.665515000
H	-4.449337000	2.546510000	-0.046120000
O	-1.567335000	-1.986442000	1.856957000
H	-0.622586000	-3.270670000	0.689580000
C	1.998377000	-1.081190000	-1.719754000
H	1.764794000	-0.929877000	-2.778740000
H	1.883277000	-2.141976000	-1.481289000
C	1.015489000	-0.265980000	-0.857365000
C	1.255672000	-0.555580000	0.708105000
H	0.368122000	-0.195235000	1.231649000
H	1.291625000	-1.641190000	0.821735000
C	2.486001000	0.128490000	1.333987000
H	2.376914000	-0.037915000	2.415529000
H	2.424800000	1.209916000	1.179683000
H	3.037034000	-0.800036000	-1.547524000
C	3.839291000	-0.383127000	0.895424000
C	4.191888000	-1.727792000	1.088636000
C	4.770688000	0.470820000	0.289545000
C	5.434270000	-2.208338000	0.674057000
H	3.483525000	-2.403943000	1.562031000
C	6.016810000	-0.005956000	-0.126841000
H	4.511473000	1.514950000	0.133601000
C	6.351589000	-1.348776000	0.060911000
H	5.688084000	-3.253908000	0.829892000
H	6.723777000	0.672115000	-0.598350000
H	7.319006000	-1.722655000	-0.264142000
C	1.040697000	1.248887000	-1.096921000
C	2.134982000	1.897680000	-1.682622000
C	-0.053899000	2.019195000	-0.679042000
C	2.134816000	3.287097000	-1.843563000
H	2.997056000	1.329486000	-2.013782000
C	-0.054309000	3.404696000	-0.836241000
H	-0.910274000	1.525313000	-0.234239000
C	1.042844000	4.045874000	-1.419514000
H	2.992466000	3.772591000	-2.302386000
H	-0.913466000	3.981431000	-0.503110000
H	1.044915000	5.125754000	-1.543009000
O	-0.251727000	-0.773024000	-1.003490000
O	-0.195217000	-3.544143000	-0.143213000
H	-0.263306000	-2.722816000	-0.666234000

TS 4-5 (doublet, G = -1347.0587)

C	-3.801738000	2.868710000	-0.542929000
C	-2.969275000	2.655410000	-1.649216000
C	-2.473113000	1.380814000	-1.936492000
C	-2.828225000	0.318822000	-1.097078000
C	-3.697966000	0.534200000	0.016590000
C	-4.177351000	1.805065000	0.295518000
H	-4.167400000	3.869803000	-0.331806000
H	-2.700115000	3.491482000	-2.288394000
H	-1.825676000	1.222987000	-2.789160000
H	-4.829705000	1.979316000	1.146916000
C	-2.450147000	-1.078597000	-1.097501000
C	-3.140810000	-1.733090000	-0.003779000
C	-3.114493000	-3.064712000	0.417144000
C	-3.864530000	-3.417926000	1.542979000
C	-4.616176000	-2.455057000	2.232635000
C	-4.637496000	-1.114064000	1.815678000
C	-3.899325000	-0.753986000	0.696117000
H	-2.526078000	-3.803031000	-0.119706000
H	-3.864495000	-4.447670000	1.889079000
H	-5.190585000	-2.750310000	3.106429000
H	-5.218703000	-0.376650000	2.362703000
O	-1.637050000	-1.725309000	-1.882058000
H	-0.960000000	-1.148107000	-2.441579000
C	2.713649000	0.662721000	-1.794941000
H	2.655225000	1.670005000	-2.220103000
H	2.750047000	-0.054207000	-2.619601000
C	1.460532000	0.372930000	-0.962448000
C	1.426006000	-1.082821000	-0.421681000
H	0.381978000	-1.318354000	-0.192773000
H	1.720746000	-1.749383000	-1.239292000
C	2.265804000	-1.389780000	0.836750000
H	2.044667000	-2.427393000	1.121071000
H	1.918676000	-0.761029000	1.664044000
H	3.635875000	0.572605000	-1.217595000
C	3.762588000	-1.232131000	0.676760000
C	4.482160000	-2.081201000	-0.177980000
C	4.460847000	-0.222247000	1.352396000
C	5.855698000	-1.918176000	-0.362746000
H	3.956699000	-2.872911000	-0.707702000
C	5.836682000	-0.054088000	1.171039000
H	3.917797000	0.447157000	2.014885000
C	6.539104000	-0.900024000	0.309930000
H	6.394120000	-2.585730000	-1.031138000
H	6.357738000	0.738958000	1.701881000
H	7.608646000	-0.770593000	0.165561000
C	1.210212000	1.390036000	0.166976000
C	2.126292000	2.398089000	0.493896000
C	0.009619000	1.310926000	0.895719000
C	1.856639000	3.294109000	1.533341000
H	3.062692000	2.484272000	-0.045527000
C	-0.264295000	2.210349000	1.923657000
H	-0.716580000	0.547228000	0.644280000
C	0.661221000	3.207445000	2.248969000
H	2.585135000	4.062566000	1.779304000
H	-1.202289000	2.133752000	2.467615000
H	0.451655000	3.908914000	3.052300000
O	0.310420000	0.625149000	-1.757427000
O	0.240450000	-0.603378000	-3.064260000
H	0.056693000	0.094285000	-3.717546000

6 (doublet, G = -310.230996)

C	-2.82345600	-0.33481300	0.55871900
H	-3.85557600	-0.57291700	0.31758200
H	-2.48671100	-0.49269700	1.57950300
C	-1.97050900	0.41343500	-0.41393100
H	-2.18455300	1.49609600	-0.35739800
H	-2.24946400	0.12266900	-1.43833600
C	-0.48061400	0.19876300	-0.21326700
C	0.39056300	1.27677600	-0.01236500
C	0.05411700	-1.09902400	-0.22756400
C	1.76153700	1.06818200	0.16617400
H	-0.00763800	2.28903800	0.00546600
C	1.42168400	-1.31231200	-0.05044000
H	-0.61240400	-1.94556900	-0.37781300
C	2.28189800	-0.22737000	0.14750100
H	2.42074600	1.91861600	0.32196800
H	1.81703400	-2.32502100	-0.06673100
H	3.34725200	-0.39204100	0.28680100

7 (doublet, G = -384.956506)

C	2.55327700	1.04976100	-0.00098900
H	2.34194600	1.66410500	-0.88453200
H	3.60718500	0.76376300	-0.00131700
C	1.69993500	-0.20218000	0.00013900
H	2.34283300	1.66523100	0.88197800
C	0.20748300	-0.05713800	0.00017000
C	-0.42840600	1.19508200	0.00057700
C	-0.57847000	-1.22201900	-0.00034000
C	-1.82158000	1.27805400	0.00045900
H	0.15597900	2.10950000	0.00102900
C	-1.96798000	-1.13884600	-0.00054900
H	-0.07895500	-2.18562000	-0.00064400
C	-2.59290400	0.11319700	-0.00014400
H	-2.30425900	2.25154900	0.00081800
H	-2.56613400	-2.04610600	-0.00104600
H	-3.67771700	0.17970600	-0.00030700
O	2.21887400	-1.31219900	0.00101000

TS 5 – 6 and 7 (doublet, G = -695.151172)

C	-0.09406600	1.41427700	-1.69322800
H	0.33568200	1.21802400	-2.68462300
H	-0.62171400	2.37058300	-1.73148700
C	1.05408200	1.52582000	-0.68678500
C	0.03941300	1.69722900	1.08415500
H	0.87307000	2.05313500	1.68483800
H	-0.63418800	2.49314300	0.77359500
C	-0.59368200	0.42808600	1.59555500
H	-0.88328400	0.65668100	2.63758100
H	0.16006400	-0.36278000	1.66924200
H	-0.80703900	0.62038300	-1.46799800
C	-1.81338200	-0.07189100	0.85047200
C	-2.92583500	0.75991700	0.65233500
C	-1.85858000	-1.37903200	0.34826500
C	-4.04573400	0.30284100	-0.04340600
H	-2.91351200	1.77458400	1.04384300
C	-2.97879900	-1.84124500	-0.34835100
H	-1.00522200	-2.03555200	0.49603300
C	-4.07502800	-1.00023200	-0.55006200

H	-4.89687600	0.96322300	-0.18914500
H	-2.99202200	-2.85756600	-0.73397200
H	-4.94619400	-1.35625500	-1.09373800
C	1.84950300	0.26913900	-0.38679900
C	1.44865300	-1.00185600	-0.82362900
C	3.03452100	0.38527900	0.35625300
C	2.21505700	-2.13079400	-0.52036800
H	0.53822200	-1.12104200	-1.40037700
C	3.79544100	-0.74013200	0.66518400
H	3.34663100	1.37292900	0.68139700
C	3.38726500	-2.00526000	0.22769700
H	1.89290900	-3.10824400	-0.87047000
H	4.70941300	-0.63304800	1.24402900
H	3.98006500	-2.88422900	0.46716000
O	1.65387700	2.64539500	-0.61120600

8 (singlet, G = -386.132676)

C	-2.44416300	-0.42463600	-0.34582800
H	-3.42658600	-0.84108000	-0.06948400
H	-2.11244500	-0.93564100	-1.26369100
C	-1.44736100	-0.70014000	0.78214600
H	-1.50808300	-1.76669100	1.03040000
H	-1.77657300	-0.14050400	1.66675700
C	-0.02436600	-0.33042700	0.42533000
C	0.42073300	0.99656900	0.52255900
C	0.87070400	-1.30080400	-0.04691300
C	1.72362300	1.34291900	0.15967000
H	-0.26564000	1.75940500	0.87857500
C	2.17572700	-0.95903200	-0.41166500
H	0.54223500	-2.33518000	-0.12699700
C	2.60695700	0.36564500	-0.30895600
H	2.05064100	2.37650700	0.24398300
H	2.85506800	-1.72781000	-0.77182300
H	3.62248700	0.63431300	-0.58861100
O	-2.52547400	0.98673300	-0.54083000
H	-3.06842600	1.14225400	-1.33053200

9 (singlet, G = -460.228361)

C	-2.15197800	-0.10286100	0.18789500
C	-1.15914400	0.50061000	-0.79077700
H	-1.37749000	1.57123700	-0.87074700
H	-1.39188200	0.06866400	-1.77342600
C	0.28329800	0.25804100	-0.41956500
C	0.84922800	-1.01600800	-0.56684600
C	1.07776100	1.29536600	0.08224900
C	2.18064100	-1.24760900	-0.22023300
H	0.23964500	-1.82887600	-0.95404800
C	2.41235700	1.06772300	0.42960500
H	0.64936100	2.28805600	0.20107600
C	2.96722800	-0.20479900	0.27940700
H	2.60550400	-2.24082800	-0.34140300
H	3.01646400	1.88485200	0.81558500
H	4.00532200	-0.38374400	0.54722600
O	-1.89701000	-0.83038000	1.12314500
O	-3.41491100	0.26776800	-0.12768300
H	-4.00789100	-0.16123700	0.52163600

End of Supporting Information
